# MouseView.js: Reliable and valid attention tracking in web-based experiments using a cursor-directed aperture

**DOI:** 10.3758/s13428-021-01703-5

**Published:** 2021-09-29

**Authors:** Alexander L. Anwyl-Irvine, Thomas Armstrong, Edwin S. Dalmaijer

**Affiliations:** 1 MRC Cognition and Brain Sciences Unit, University of Cambridge, Cambridge, UK; 2 Department of Psychology, Whitman College, Walla Walla, WA USA; 3 School of Psychological Science, University of Bristol, Bristol, UK

**Keywords:** Attention, eye tracking, online experiments, JavaScript, open-source, cyberpsychology

## Abstract

Psychological research is increasingly moving online, where web-based studies allow for data collection at scale. Behavioural researchers are well supported by existing tools for participant recruitment, and for building and running experiments with decent timing. However, not all techniques are portable to the Internet: While eye tracking works in tightly controlled lab conditions, webcam-based eye tracking suffers from high attrition and poorer quality due to basic limitations like webcam availability, poor image quality, and reflections on glasses and the cornea. Here we present MouseView.js, an alternative to eye tracking that can be employed in web-based research. Inspired by the visual system, MouseView.js blurs the display to mimic peripheral vision, but allows participants to move a sharp aperture that is roughly the size of the fovea. Like eye gaze, the aperture can be directed to fixate on stimuli of interest. We validated MouseView.js in an online replication (*N* = 165) of an established free viewing task (*N* = 83 existing eye-tracking datasets), and in an in-lab direct comparison with eye tracking in the same participants (﻿N = 50). Mouseview.js proved as reliable as gaze, and produced the same pattern of dwell time results. In addition, dwell time differences from MouseView.js and from eye tracking correlated highly, and related to self-report measures in similar ways. The tool is open-source, implemented in JavaScript, and usable as a standalone library, or within Gorilla, jsPsych, and PsychoJS. In sum, MouseView.js is a freely available instrument for attention-tracking that is both reliable and valid, and that can replace eye tracking in certain web-based psychological experiments.

**Supplementary Information:**

The online version contains supplementary material available at 10.3758/s13428-021-01703-5.

## Introduction

Encouraged by the ever-increasing accessibility and quality of tools for web-based experimentation, psychological researchers have gradually moved behavioural and self-report studies online (see Anwyl-Irvine, Dalmaijer, et al., 2020a, for a brief history). One technique that has yet to make this transition is eye tracking, a popular method that yields rich data on gaze fixation patterns, saccade dynamics, and pupil size (Holmqvist et al., 2015). While webcam-based eye tracking was a relatively niche topic in cognitive and behavioural sciences until recently, pandemic-related lockdowns and related lab closures have caused a sudden surge in interest.

Modern approaches to webcam eye tracking typically employ a combination of facial landmark detection (Kazemi & Sullivan, 2014; Saragih et al., 2011), sometimes aided by pupil detection (Papoutsaki et al., 2015), and (regularised) regression models to map landmarks to gaze positions (Papoutsaki et al., 2015; Xu et al., 2015). Others have taken a slightly more complex approach, in which extracted features (e.g. each eye, the face, facial location within the image) were cropped from the webcam stream, and passed through a neural network (Krafka et al., 2016; Meng & Zhao, 2017). Many algorithms for sub-components of eye and gaze detection cascades have been proposed, see Gómez-Poveda and Gaudioso (2016) for a summary and evaluation.

One currently popular and accessible package, built as an extension of TurkerGaze (Xu et al., 2015), is WebGazer (Papoutsaki et al., 2015). When independently tested under ideal conditions (e.g. high-resolution webcam, clean camera, no reflections on the eye that obscure the pupil, participant not too far or too close, none to low participant movement), WebGazer produces reasonable accuracy and precision (about 17% of screen size, which translates into variable size in degrees of visual angle due to participants’ non-standardised home environments) (Semmelmann & Weigelt, 2018). It is good enough for studies that need only rough gaze estimation, and thus cause for optimism among many.

Unfortunately, when employed “in the wild”, webcam-based eye-tracking suffers from high attrition (participants who start the study, but fail the eye-tracking calibration): 62% in Semmelmann and Weigelt (2018) and 61% in Yang and Krajbich (2020). This is not due to operator error: both studies were conducted by able programmers who adapted the WebGazer source code for their own purposes. Instead, participants were likely unable to pass the calibration procedure due to poor image quality, suboptimal lighting conditions, and reflections on the cornea or their glasses. In addition, the technique obviously excludes participants who do not own a webcam. In sum, samples in web-based eye-tracking are biased by definition (because over 60% of participants who try to take part fail the calibration), and suffer from heavy attrition due to fundamental limitations on eye signal in webcam streams.

Here, we describe a method that is designed to simulate gaze tracking without the need for a webcam. We achieve this by mimicking the visual system’s peripheral blur and foveal clarity; specifically by allowing participants to move a high-fidelity aperture on an otherwise obscured field with their mouse. The result is akin to providing participants with a narrow-beam torch, and placing them in a dark room in which the experimenter has arranged the furniture in a particular way.

We are by no means the first to suggest such a technique. In fact, the idea of limiting viewing with a gaze-contingent aperture is almost half a century old (McConkie & Rayner, 1975). It has been used profitably in reading research (Rayner, 2014), and to simulate the viewing behaviour of macular degeneration patients (Lingnau et al., 2008, 2010) who develop “preferred retinal locations” that act somewhat similarly to the fovea (Bethlehem et al., 2014). Decoupling eyes and viewing aperture is at least two decades old, when apertures were locked to computer mouse cursors rather than gaze (Blackwell et al., 2000; Jansen et al., 2003), or gaze was limited to randomly placed (Gosselin & Schyns, 2001) or participant-operated(Deng et al., 2013) “bubbles” (perforations in a blurring mask through which participants could view the underlying stimulus material).

More recent work has taken both the bubble (Deng et al., 2013; Kim et al., 2017, 2015) and the moving aperture approaches (Gomez et al., 2017; Jiang et al., 2015) to the Internet, usually with a view to investigate specific types of stimuli (e.g. data visualisations) or to accrue large datasets for the development of visual saliency models. Aside from producing excellent names (“*Fauxvea*” by Gomez et al., 2017), these efforts have been particularly successful in demonstrating that mouse-guided visual exploration overlaps relatively closely with free viewing patterns.

While highly encouraging, the cited work has not produced readily available software for psychological experiments (although similar code snippets exist, e.g. this PsychoPy demonstration: **gitlab.pavlovia.org/demos/dynamic_selective_inspect**). In addition, it remains unclear whether non-saliency aspects of gaze behaviour are equally well approximated by the mouse-locked aperture paradigm. We address both of these issues by presenting an open-source JavaScript library, MouseView.js, and by testing this in free viewing experiments that probe more than just visual saliency. MouseView.js can be used as a standalone library, and is integrated into popular experiment-building platforms Gorilla (www.gorilla.sc, Anwyl-Irvine, Massonnié, et al., 2020b), jsPsych (www.jspsych.org, de Leeuw, 2015), and PsychoPy/PsychoJS (www.psychopy.org, Peirce et al., 2019).

We present two validation studies, in which we compare MouseView.js and eye tracking in preferential looking experiments. The first validation study replicated an existing eye-tracking study (Armstrong et al., 2020) in a web-based experiment, and we compared data between the original and our online sample. In a second validation study, we recruited a new sample to take part in two lab-based experiments to directly compare gaze and mouse within the same participants.

## MouseView.js

At the core of MouseView.js is a highly configurable JavaScript library. This obscures a webpage with an overlay, permits the user to view the page through an aperture, and records coordinates of the mouse cursor or screen-touches. The library is built to be as flexible as possible. It can create an overlay over an online experimental task (as we demonstrate in our validation study), or it can be used on a dynamic website for user-experience research. The overlay does not prevent user interaction, so users can click or press buttons, and type on the keyboard as usual.

In this section, we give an overview of the available configuration options and methods. For a more complete and up-to-date overview, including some tutorials on implementation, we recommend reading our documentation at www.mouseview.org/docs. For the purposes of this description, we will use the term *user* to describe the participant or page viewer, and the term *researcher* to describe the person setting up MouseView.js.

### Mechanism

The library is designed using the relatively recent ES6 Module architecture. This means it can be included in an existing website or app with minimal scripting. Once included in a webpage, the library creates a globally accessible object with variables and functions that any piece of code should be able to access. This object is called *mouseview,* and it contains all the methods needed to produce an overlay and to track mouse movements. This is architecture is analogous to other libraries, like WebGazer.js (Papoutsaki et al., 2015).

### Configuration

**Fig. 1:**
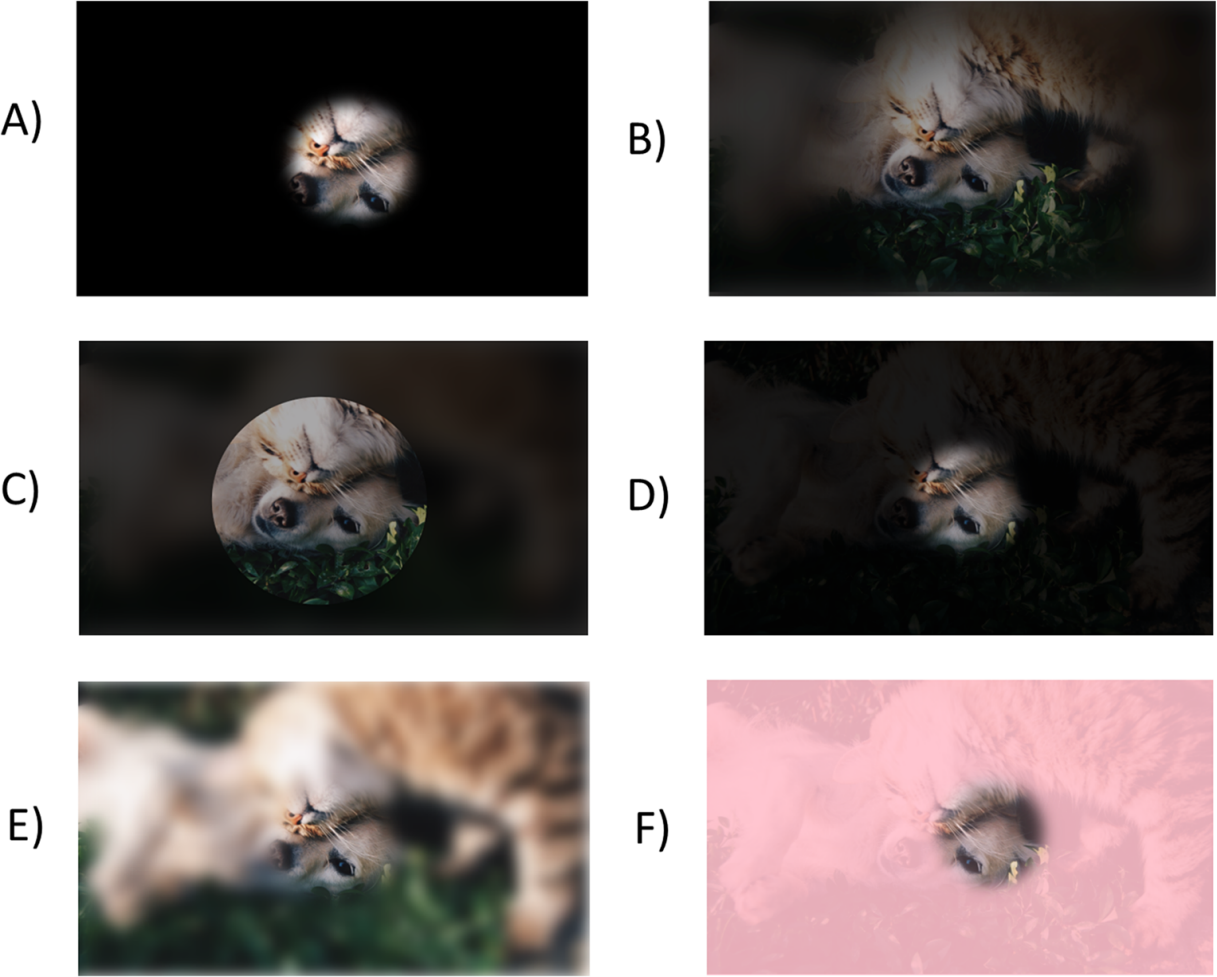
Screenshots of different MouseView.js configurations. **a** Solid black overlay with Gaussian Edge SD of 5 pixels (overlayColour=’black’, overlayAlpha=1, overlayGaussian=0, apertureGauss=5). **b** Gaussian overlay and Gaussian aperture edge with SD of 50 pixels (overlayColour=’black’, overlayAlpha=0.8, overlayGaussian=20, apertureGauss=50). **c** Gaussian overlay with solid aperture edge (overlayColour=’black’, overlayAlpha=0.8, overlayGaussian=20, apertureGauss=0). **d** No Gaussian blur but overlay with 0.9 alpha opacity (overlayColour=’black’, overlayAlpha=0.9, overlayGaussian=0, apertureGauss=10). **e** Gaussian blurred overlay with 0.0 opacity (overlayColour=’black’, overlayAlpha=0, overlayGaussian=20, apertureGauss=10). **f** Pink overlay (overlayColour=’#FF69B4’, overlayAlpha=0.8, overlayGaussian=0, apertureGauss=10)

As alluded to above, there are several options that researchers can choose to set. These pertain to three main areas: aperture, overlay, and recording. These are customised by specifying variables in the *mouseview.params* object*.* These variables are summarised in Table 1, and Fig. 1 illustrates some of the possible configurations.

**Table 1:** Configuration options, description and default values for MouseView.js

Setting	Description	Accepted types	Default
Aperture			
mouseview.params.apertureSize	Size of the viewing aperture in pixels or percentage of screen width.	Number-Integer (pixels) or String (‘x%’)	‘5%’
mouseview.params.apertureGauss	Standard Deviation for Gaussian edge.	Number-Integer (pixels)	10
Overlay			
mouseview.params.overlayColour	Colour of overlay.	String containing CSS Keyword, hexadecimal, or HSL code.	‘black’
mouseview.params.overlayAlpha	Transparency of overlay.	Number-Decimal (0-1).	0.8
mouseview.params.overlayGaussian	Standard Deviation for Gaussian blurring of overlay.	Number-Integer (pixels)	20
mouseview.params.overlayGaussianFunc	Callback function that will be run when the Gaussian overlay has been generated.	JavaScript arrow function. “() => {}”	() => {console.log('overlay generated')}
mouseview.params.overlayGaussianInterval	Millisecond Interval for the Gaussian blur overlay to be regenerated. Passing 0 means it will only update on window resize and page scroll.	Number-Integer or Float (milliseconds)	0
Recording			
mouseview.timing.sampleRate	Desired target sample rate. “Target” because timing linked to screen refreshes can be variable.	Number-Integer or Float (milliseconds)	16.66 (one refresh at 60 Hz)

#### Aperture

Researchers can specify the size of the viewing aperture in pixels, or as a percentage of screen width. Specifying size as a percentage ensures that scaling is consistent across devices of different screen sizes, whilst specifying size as pixels offers a greater level of control. The relevant name of the setting is *mouseview.params.apertureSize.* It can be a string in the format ‘X%’ for a percentage scale or as an integer to specify pixel. The default setting is 5%. For most researchers, we recommend the use of the percentage option, as this will ensure a reasonable level of consistency across participants. Some restriction of browser window sizes in experimental context is sensible when using the percentage setting; as small window sizes will lead to a very small aperture. This is possible in most experiment builders.

The edge of the aperture can also be blurred. The purpose of this is to roughly simulate the edges of the foveated area (Reisfeld et al., 1995), and to avoid the potentially distracting peripheral motion effects of a solid edge (Lingnau et al., 2008). We implement a Gaussian blur here, with the researcher specifying the standard deviation of the Gaussian function in pixels. The relevant setting is *mouseview.params.apertureGauss.* Currently, this is specified as an integer only, with 0 representing a solid edge (i.e. no blur).

#### Overlay

The overlay configuration allows researchers to customise the attributes of the obscuring layer, which the aperture cuts through. The overlay is a HTML canvas that we insert into the webpage over all the content, and it is drawn based on the settings. Firstly, the overlay can be transparent or any colour supported by the cascading style sheet language (label: e.g. ‘green’, hexcode, RGB or HSL code). If a colour is specified, the transparency can be set in the form of an alpha value between 0 (fully opaque) and 1 (fully transparent).

The library’s most complex feature is the ability to dynamically blur the contents of anything that is already on a webpage. This can be done by passing a non-zero value to *mouseview.params.overlayGaussian.* The value entered here represents the standard deviation of the Gaussian kernel applied to the underlying page content. This is not straightforward, as there is currently no way to apply a blurring filter to an entire webpage, while also allowing a cut-out. MouseView.js achieves this by taking a screenshot of the webpage, applying Gaussian blur to the screenshot, and then drawing this blurred image onto the overlay. The screenshot step is computationally intensive, and uses open-source library *html2canvas (**https://github.com/niklasvh/html2canvas**).* This renders the entire webpage off-screen, and turns it into an image.

This process takes time, so we provide the option for the researcher to pass in a callback function, which will be executed once the overlay has been generated. The researcher can use this to hide the contents of the experiment whilst they are being blurred, to avoid an unobscured preview. This function can be passed into *mouseview.params.overlayGaussianFunc*, which is then executed on the blur rendering (using a JavaScript object called a *Promise*). We recommend using the arrow syntax to define these functions (i.e. “() => {}”), as this ensures the given function will have access to external variables in the browser environment. Up-to-date examples on how to implement this can be found on the documentation website (www.mouseview.org).

Lastly, we also provide the ability to control when this blur overlay is updated. It should not be done every frame, as it is a computationally heavy process. The default behaviour is thus to only update the screenshot on window resizing and scrolling events (i.e. when the page changes with a user interaction). Researchers looking to blur a video or animation are likely better of opting for an opacity filter. However, if they do opt for Gaussian blur, we provide a refresh interval setting: Specifying a non-zero value for *mouseview.params.overlayGaussianInterval,* will tell MouseView.js to regenerate the blur at a set interval. Some fast computers might manage a sub-second refresh rate, but we recommend using an interval no shorter than 1–2 s, to avoid crashing the web page.

#### Recording

MouseView.js’ recording functionality is discussed in greater detail below. Here, we highlight one optional setting that configures the sampling rate of the mouse tracking. The *mouseview.timing.sampleRate* parameter sets a target sampling rate for MouseView.js to record the mouse positions at, i.e. how many (x,y) mouse coordinates will be recorded per second (defined as the time between each sample in milliseconds). The sampling rate is constricted by the animation refresh rate, which defines where the aperture is. Slower computers or higher computational loads will reduce the consistency of the sampling rate, which is why we describe it is a ”*target* sampling rate”. The default value is 16.66 ms, which corresponds to a single screen refresh on a 60-Hz monitor.

### Functions

MouseView.js provides several functions (also called “methods”, as they are object-bound functions) to control elements on screen, and the recording of data. Like the configuration variables above, the methods are accessed through the global *mouseview* object. Table 2 gives a summary of all of these methods.

**Table 2:** Overview of methods available via the mouseview object

Method	Description
mouseview.init()	This function initiates MouseView.js with the current settings.
mouseview.removeAll()	This function removes any overlays generated by MouseView.js
mouseview.startTracking()	This starts the recording of the current mouse (or touch) coordinates, in intervals specified by the *mouseview.timing.sampleRate* variable.
mouseview.stopTracking()	This stops the recording.
mouseview.logEvent(event_txt)	A utility function that will log a string (event_txt) in the sample data, along with a timestamp, whenever it is called.
mouseview.storeData()	A utility function that stores all the current mouse-tracking data in the browser’s local storage, and automatically appends the current webpage path to the data. It will overwrite any currently stored data.
mouseview.getData()	Retrieves any stored data, and appends the current page. New data is added to this object.
mouseview.clearData()	Clear working data.
mouseview.updateOverlayCanvas()	Forces the overlay to be updated, and recaptures the screenshot if the Gaussian blur setting is in use.

The functions *Mouseview.init()* and *mouseview.removeAll(),* render the overlay and aperture or remove them, respectively. The functions *mouseview.startTracking() & mouseview.stopTracking()* control the recording of mouse movement (or screen touches) in pixel coordinates.AThe function *mouseview.getData()* returns a list of coordinates and associated timestamps, which can be piped into the format that the experimenter prefers for saving data. These are the core functions required for operating the library.

We also provide additional utility functions, including those needed for data persistence across webpages. This is particularly helpful for those wishing to conduct multi-page user experience research. By storing data with the browsers *localStorage* object, data can be passed between separate webpages. MouseView.js automatically detects and adds on the path of the page between these sessions. Persistence is achieved with the *mouseview.storeData()* and *mouseview.getData()* functions. Researchers can also log custom events (sometimes referred to as “triggers”), with timestamps relative to recorded samples. This functionality can be used to log dynamic events, and investigate mouse data relative to these events. Further utility functions are illustrated in Table 2.

## Between-participants validation study

Plenty of studies have established that the cursor-locked apertures produce exploration behaviour that resembles gaze behaviour during free viewing (Blackwell et al., 2000; Gomez et al., 2017; Jiang et al., 2015). The purpose of the current validation study was to extend previous validation efforts into a preferential looking paradigm with pairs of affective stimuli. This is fundamentally different from single-stimulus free viewing, as participants generally divide their attention between the stimuli as a function of their affective qualities. In addition, stimuli are repeated over trials, thereby rendering initial differences in visual saliency increasingly less important to gaze behaviour.

### Experiment

To validate the use of a mouse-locked aperture in overt attention research, we employed an affective preferential looking task. In this type of task, two images are shown for a relatively long time (e.g. 10 s), and participants are free to view where they would like. The affective component is in the image content: one evokes a specific emotion (independently verified by self-report), whereas the other is neutral. Image pairs are repeated over several trials, while locations (left versus right) are randomised. The measure of interest is how long participants’ gaze dwells on each image.

While the pairs are roughly matched for low-level features using a classic visual saliency model (Itti et al., 1998), the long exposure times and repeated exposures ensure that cognitive and affective processes will have an effect on participants’ dwell times for each stimulus. In the lab, this method has been used to show sustained avoidance of disgust stimuli (Dalmaijer et al., 2021), and to demonstrate how these are affected by pharmacologically altered gastric state (Nord et al., 2021). In addition, the method has been used to map overt attentional bias during trials, showing sustained biases for pleasant, threat, and suicide-related images, as well as an initial short-lived bias towards disgust stimuli that is followed by sustained avoidance (Armstrong et al., 2020).

Here, we replicate two of the summarised findings, specifically for disgusting and pleasant images, which in Experiment 2 of (Armstrong et al., 2020) were the strongest elicitors of oculomotor avoidance and approach, respectively. The original gaze data (*N* = 83) is directly compared to mouse data collected with MouseView.js (*N* = 165).

#### Procedure

Trials started with a central fixation cross, where the mouse had to be placed to advance the trial. Stimuli were then presented for 10 s, followed by a 1-s blank screen, after which the next trial started. A total of five stimulus pairs per condition were each presented four times, resulting in 20 trials with disgust and 20 trials with pleasant images. In the original eye-tracking study, two further image categories were included (suicide and threat). For simplicity, we did not include those in the MouseView.js replication, and thus do not report on them here.

Stimuli were shown in pairs, with one image appearing on the left of the screen and another on the right. Stimulus pairs were kept constant, but their left/right locations were randomised. The stimuli were scaled to the “viewport” (available display within the browser) so that their width was 25% of the viewport width; and their midpoints were positioned at 33% and 66% of the viewport width, and 50% of screen height. For example, on a 1920 x 1080 display, a typical viewport would be 1920 by 937, the stimuli would be sized 480 by 360 pixels, and their midpoints would fall on (641, 469) and (1278, 469). We did not estimate or attempt to control participants’ distance from their displays, and thus real stimulus size in degrees of visual angle was unknown.

Stimulus presentation lasted for 10 s, during which the screen was blurred (Gaussian blur with the default settings reported in Table 1). Participants could move a clear aperture with (Gaussian edges to avoid a hard boundary with the blurred area) and a size of 5% of the viewport width, which are the default settings for apertureGauss and apertureSize as reported in Table 1.

#### Stimuli

There were five disgust stimuli, each depicting bodily effluvia. They depicted a man throwing up, a toilet with excrement in and around it, a toilet with throw-up in it, a man throwing up directly into a mint-green toilet, and a close photo of loose stool (type 6 on the Bristol stool chart).

Pleasant images depicted three young children, a couple enjoying a bicycle trip, an elderly couple waving to the camera on a sunny quay, several laughing children, and four children in a catalogue-type photo.

The neutral images to which the above affective image were matched portrayed a close-up of buttons (for sewing onto clothing), assorted electrical and general-purpose tools hanging on a wall, a picnic bench, an electric clothes iron, a glass mug on a table, a metal dustpan lying on a floor, a hair dryer, four clothes pins in different colours, the bottom of a candle holder that is lying down, and a wire stripper.

#### Participants

Participants were recruited via Prolific Academic, and tested via the Gorilla experimentation platform. This platform has been shown to have sufficient display timing for our purposes of relatively long stimulus displays (Anwyl-Irvine, Dalmaijer, et al., 2020a; Bridges et al., 2020).

Data were collected in September 2020. Out of 165 participants, 45% self-identified as a woman, 55% as a man, and 0% as non-binary. Their age distribution spanned from 18 to 76 years, with an average of 31.5 (median = 29) and a standard deviation of 10.9. Participants reported being born in Brazil (1.2%), Canada (50.6%), China (4.8%), Germany (0.6%), Hong Kong (2.4%), India (1.8%), Iran (0.6%), Mexico (0.6%), Nigeria (3.0%), Pakistan (1.2%), Peru (0.6%), Philippines (1.2%), Saint Luca (0.6%), South Korea (1.2%), Sweden (0.6%), Taiwan (1.2%), Ukraine (0.6%), United Kingdom (1.8%), United States (24.7%), and Venezuela (0.6%); and all lived in either Canada (75%) or the United States (25%).

The MouseView.js data was then compared with data from 83 participants who took part in an eye-tracking study that was published elsewhere (Armstrong et al., 2020). This study was skewed towards university undergraduate students at Queen’s University (Kingston, ON, Canada). Their average was 19.71 years (SD = 2.06 years); their gender identity 83.3% women, 14.3% men, and 1.2% as nonbinary; and their racial/ethnic identity 46.4% White, 36.9% Asian, 6% Indigenous, 4.8% Latino/a, 3.6% Black, and 1.2% Middle Eastern or North African.

It should be noted that details were subtly different between the original study and the current mouse-aperture replication. Due to the nature of web-based data collection, display size (and thus stimulus display) was different for each MouseView.js participant. In addition, the original eye-tracking experiment had two additional stimulus conditions (threat and suicide-related), which were omitted for simplicity in the MouseView.js experiment. Finally, while affective-neutral stimulus pairing remained constant in the MouseView.js experiment, it was not in the eye-tracking experiment. For all of these reasons, direct statistical comparisons would not be particularly informative. Heatmap and scanpath comparisons are presented as they are, and interpreted qualitatively.

### Reliability

We examined whether MouseView.js produced reliable results by computing the difference in dwell time between the affective and neutral stimulus in each trial. We then computed Cronbach’s coefficient α, and the split-half reliability as the average Spearman–Brown coefficient (ρ) over 100 different halfway-splits of all trials within each condition (disgust or pleasant).

The results are summarised in Table 3, and show that differences in dwell time between the affective and the neutral image in each trial are of good reliability for disgust stimuli for both gaze (ρ = 0.81, α = 0.78) and mouse (ρ = 0.89, α = 0.86). Dwell time difference was of lower reliability for pleasant images for both gaze (ρ = 0.54, α = 0.53) and mouse (ρ = 0.71, α = 0.69). These results indicate that reliability was similar between eye-tracking and the mouse-locked aperture experiments, and in fact slightly better for mouse-aperture dwell.

**Table 3:** The reliability of dwell-time differences between neutral and affective stimuli in 20 trials, estimated by the Spearman–Brownsplit-half reliability ρ (and the standard error of its mean across splits) over 100 random splits, and Cronbach’s coefficient alpha

Stimulus type	Eye tracking		Mouse view	
	ρ (se)	α	ρ (se)	α
Disgust	0.81 (3.44e-3)	0.78	0.89 (1.82e-3)	0.86
Pleasant	0.54 (8.20e-3)	0.53	0.71 (5.52e-3)	0.69

#### Behavioural consistency

**Fig. 2:**
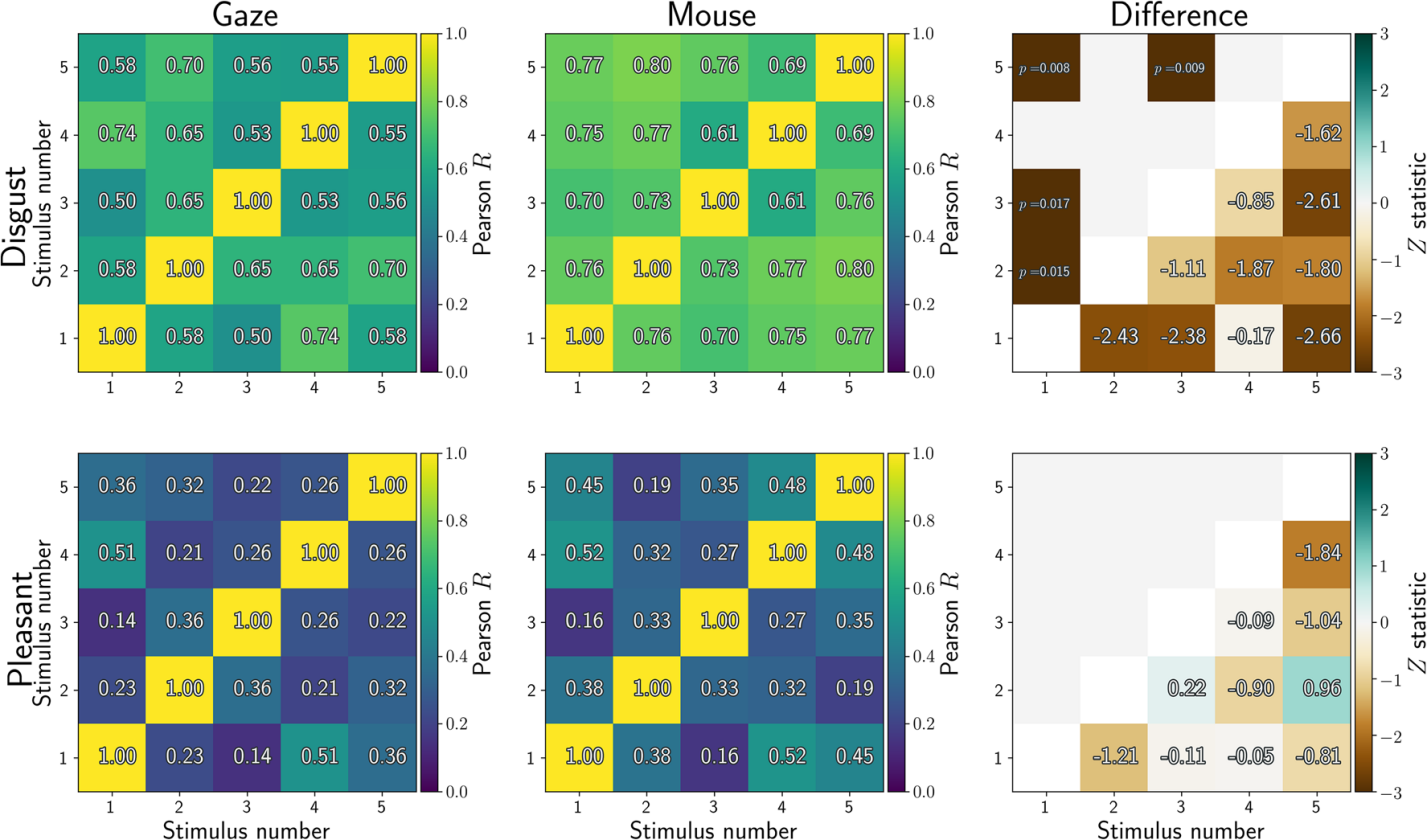
Correlations between affective-neutral dwell time differences for all stimuli (averaged across all four presentations). Higher correlations indicate that participants showed similar dwell time differences between stimuli. Included here are five disgust stimuli (*top row*) and five pleasant stimuli (*bottom row*). Dwell times were computed from eye tracking (*left column*) or MouseView.js (*middle column*), and the difference between them is reported in the right column

Related to reliability is how similarly participants respond to all images. Five different images were employed in each condition, and we computed dwell-time differences between the affective and neutral stimulus for each participant (over whole trials, then averaged across four presentations). We then correlated the dwell-time differences for each stimulus with those for all other stimuli, resulting in Fig. 2. Values close to 1 would illustrate that participants avoided (or approached) stimuli in the same way, whereas values close to 0 indicate that participants were less consistent in their gaze or mouse behaviour towards the stimuli.

Dwell differences were more consistent for disgusting compared to pleasant images, but similar between gaze and mouse aperture: Four out of twenty correlations were statistically significantly different between gaze and mouse. For these significant cells (combinations of stimulus and repetition number), mouse data were more consistent between participants compared to eye-tracking data.

**Fig. 3:**
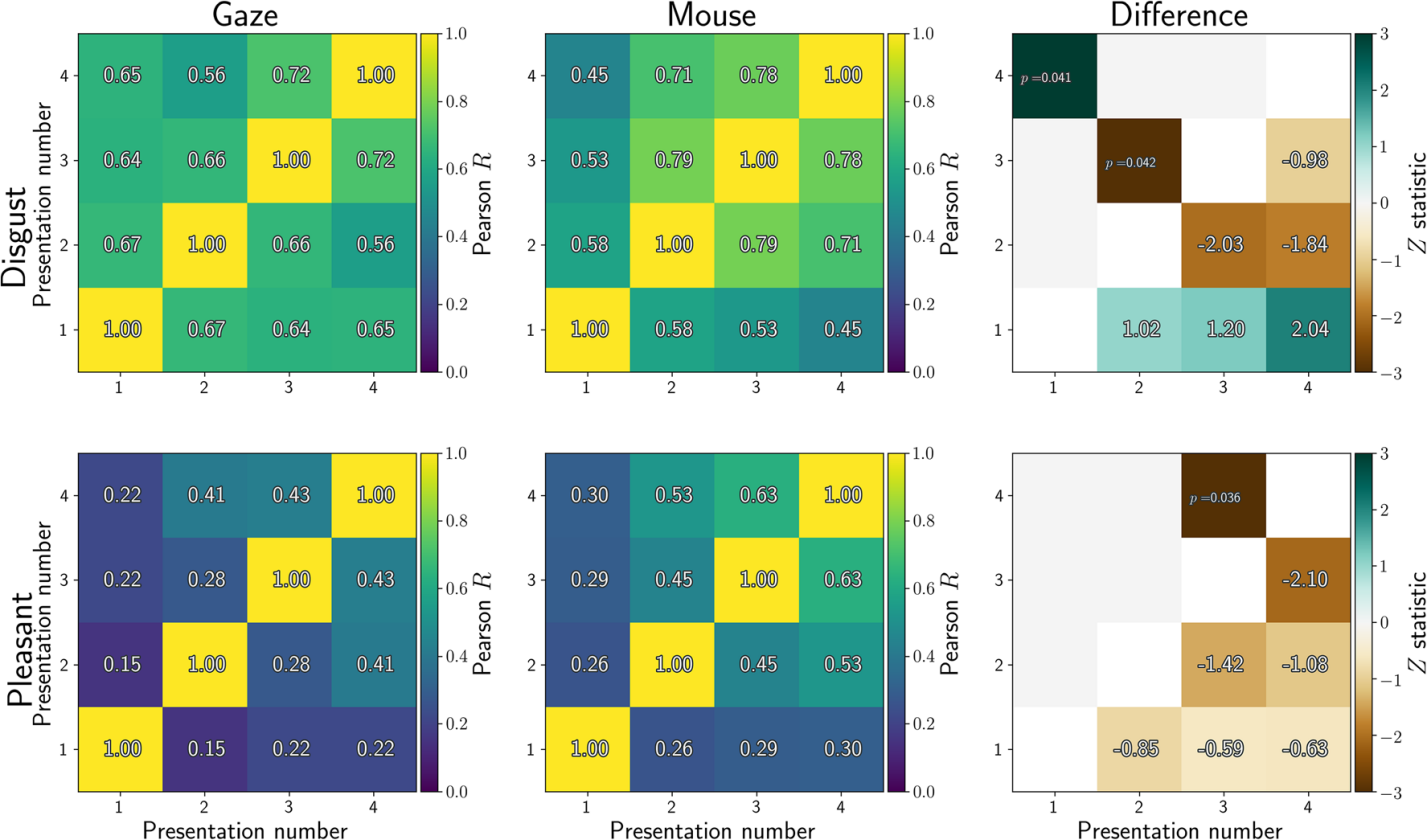
Correlations between affective-neutral dwell time differences for all repetitions of the same stimuli (averaged across all five stimuli within each condition). Higher correlations indicate that participants showed similar dwell time differences between repetitions. Included here are four repetitions of five disgust stimuli (*top row*) and five pleasant stimuli (*bottom row*). Dwell times were computed from eye tracking (*left column*) or MouseView.js (*middle column*), and the difference between them is reported in the right column

We computed similar metrics for each presentation number, averaged across stimuli within each condition. Three out of twelve correlations differed between gaze and mouse (Fig. 3).

It should be noted that one would not necessarily expect the above correlations to be high, nor that they directly reflect measurement reliability. This is because real differences exist between the first and later presentations of stimulus pairs in the current design: the first presentation of a disgust-neutral stimulus pair provokes less disgust avoidance than later presentations of the same pair (Armstrong et al., 2020; Dalmaijer et al., 2021; Nord et al., 2021).

In sum, these results show that behaviour is more stable between stimuli and presentations for disgusting compared to pleasant images. They also indicate that dwell behaviour is consistent between gaze and MouseView.js experiments, although it is more consistent in mouse recordings for a minority of stimuli, and different in consistency only between presentation numbers 1 and 4 (more consistent for gaze) and 2 and 3 (more consistent for mouse).

### Validity

The objective of the following analyses was to determine whether mouse-locked apertures genuinely track participants’ overt attention. We established this by directly comparing MouseView.js data with the common attention-tracking method of gaze tracking. Specifically, we compared scan paths and dwell-time differences between stimuli.

In addition, we correlated dwell time differences between affective and neutral stimuli with self-reported disgust and pleasantness ratings for those stimuli. It has previously been shown that the oculomotor avoidance of disgust stimuli correlates with self-reported disgust (Dalmaijer et al., 2021). We thus expected mouse dwell to show the same.

#### Pre-registered hypotheses

We pre-registered the following hypotheses, which correspond to the main eye-tracking findings in Armstrong et al. (2020): 1) Participants will view disgusting images less overall compared to accompanying neutral images. 2) Participants will view pleasant images more overall compared to accompanying neutral images. 3) Participants' disgust ratings of the disgusting images will correlate negatively with their overall viewing time of the disgusting images. 4) Participants' pleasantness ratings of the pleasant images will correlate positively with their overall viewing time of the pleasant images. 5) Both disgusting and pleasant images will initially capture "attention" (gaze directed at the image) relative to the neutral image. Then disgusting images will be viewed less as the trial progresses (eventually less than the accompanying neutral image), whereas pleasant images will continue to be viewed more than the neutral image. 6) Dwell on the disgusting image will be greatest on the first exposure to an image, and then will decrease once the disgusting image becomes familiar. 7) Disgust ratings of disgust images will be associated with a greater slope of decreasing viewing across trials. The full pre-registration can be found at https://osf.io/mta2d.

#### Comparing mouse and gaze dwell time

**Fig. 4:**
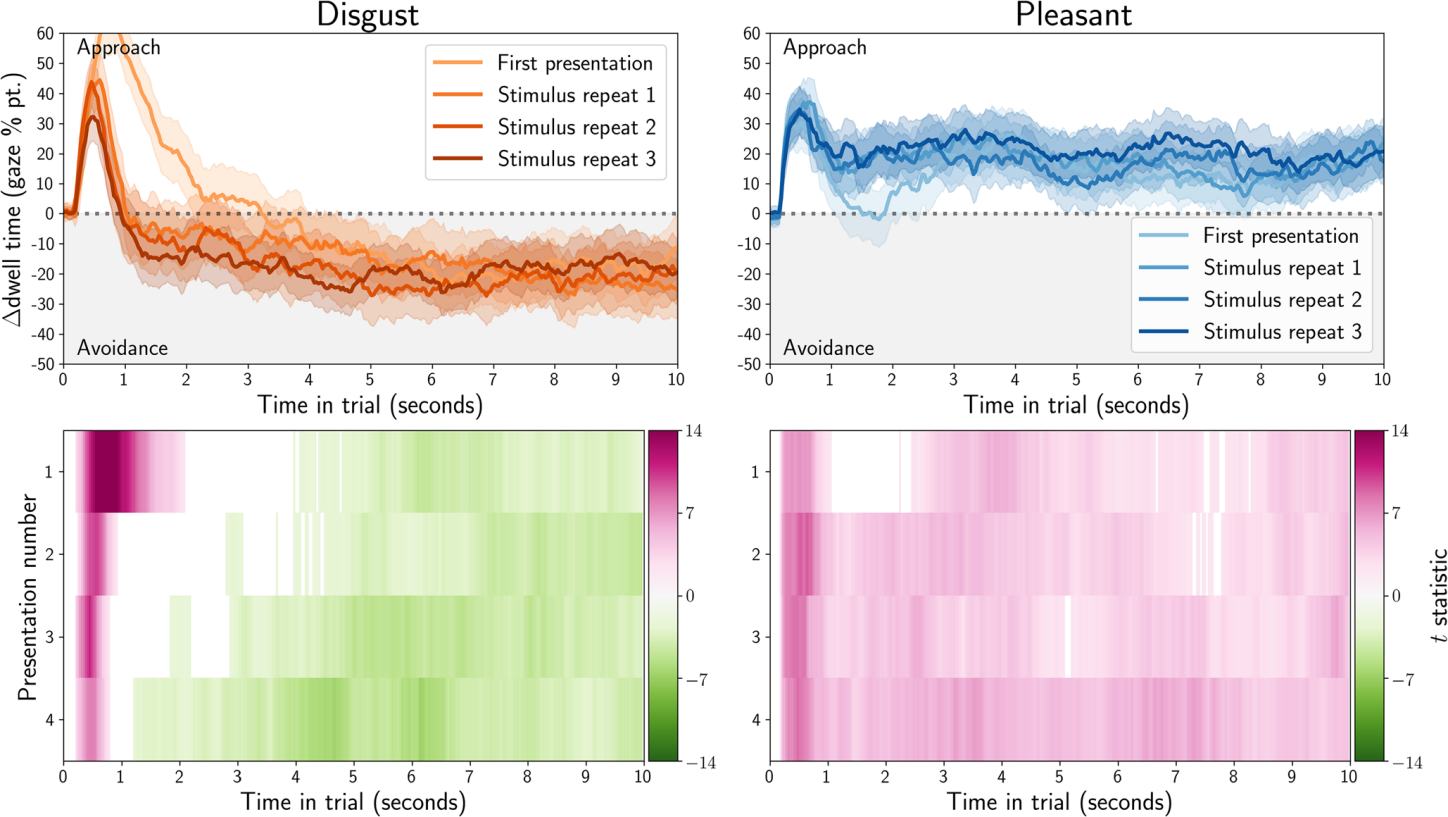
Gaze dwell time difference (in percentage points) between affective and neutral stimuli as obtained in an eye-tracking task. Positive values indicate participants spent more time looking at the affective (disgust or pleasant) stimulus than the control stimulus; negative values indicate the opposite. In the *top row*, *solid lines* indicate averages and *shading* the within-participant 95% confidence interval. In the *bottom row*, *t* values from one-sample*t* tests of the dwell difference against 0 are reported, but only for those tests where *p* < 0.05 (uncorrected)

**Fig. 5:**
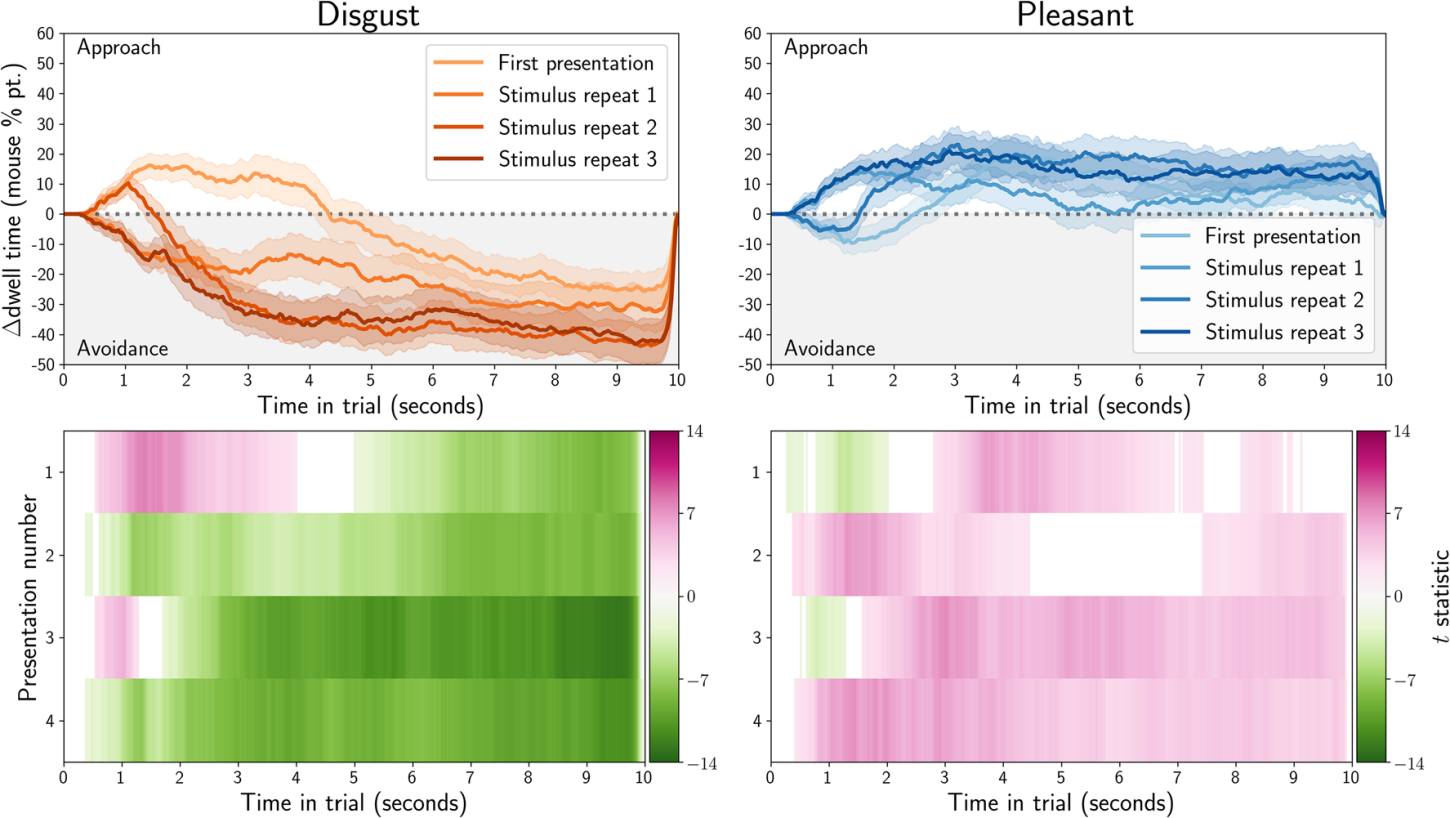
Mouse dwell time difference (in percentage points) between affective and neutral stimuli as obtained in a MouseView.js task. Positive values indicate participants spent more time looking at the affective (disgust or pleasant) stimulus than the control stimulus; negative values indicate the opposite. In the *top row*, *solid lines* indicate averages and *shading* the within-participant 95% confidence interval. The sharp return to 0 at the end of the trial duration is an artefact of mouse recording cutting out slightly too early. In the *bottom row*, *t* values from one-sample*t* tests of the dwell difference against 0 are reported, but only for those tests where *p* < 0.05 (uncorrected)

Dwell time was computed as the total duration of gaze or mouse samples for which the coordinate was within an image. The difference in dwell time for affective and neutral stimuli was computed between each of the stimulus pairs, and then averaged over all stimuli within each condition (disgust or pleasant). We also computed one-sample*t* tests between the dwell time for the affective (disgust or pleasant) and neutral stimulus, after averaging dwell times over all stimuli within each condition (disgust or pleasant). The results are plotted in the bottom rows of Fig. 4 for gaze, and Fig. 5 for MouseView.js.

These results indicate that participants showed sustained bias towards pleasant stimuli, and away from disgust stimuli. The exception to this is an initial bias towards disgust stimuli, which is particularly apparent in the eye-tracking experiment, but also in the first trial of the MouseView.js data.

**Fig. 6:**
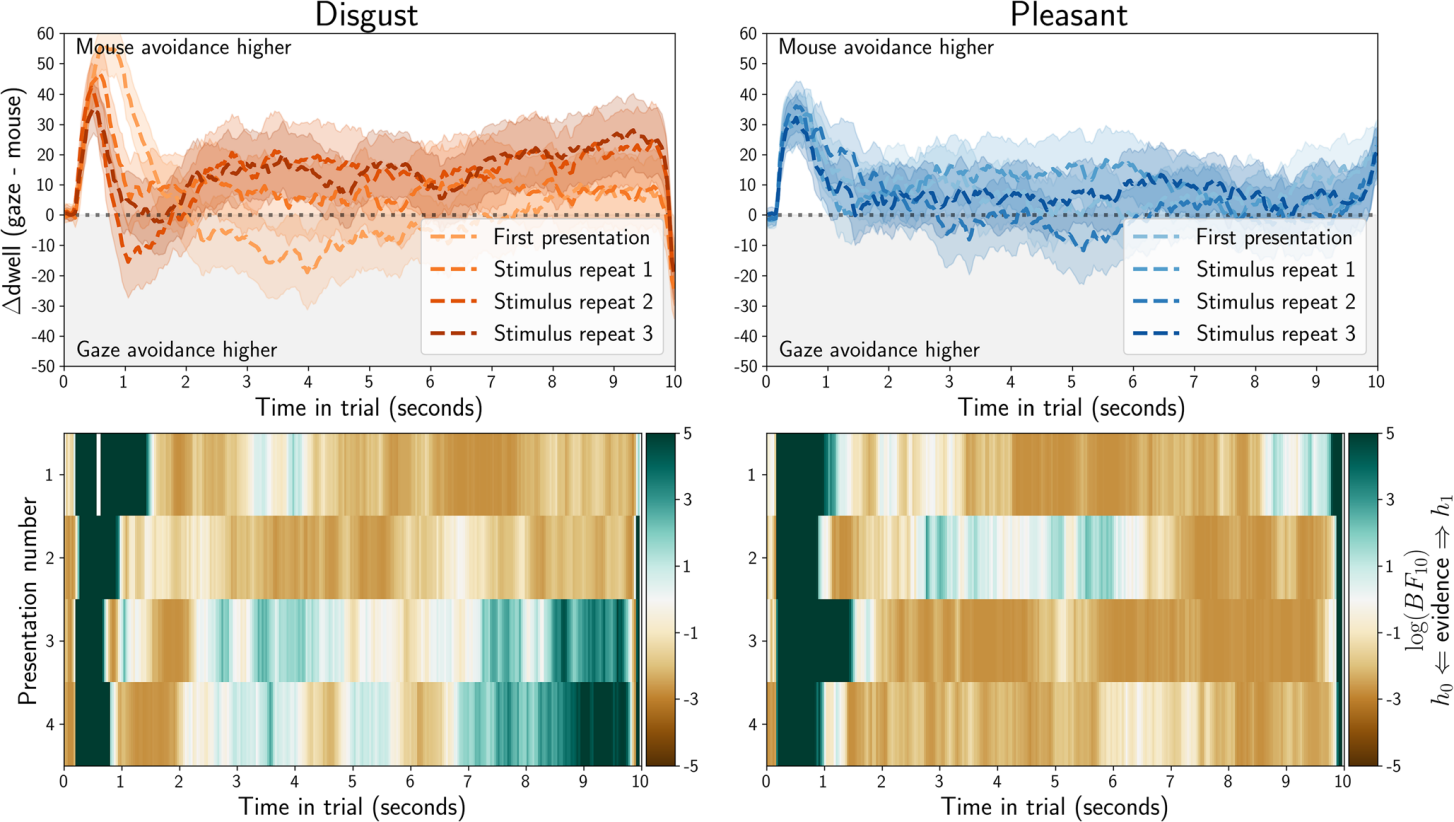
Quantification of the difference between gaze dwell time differences (eye tracking, Fig. 4) and mouse dwell times (MouseView.js, Fig. 5). Positive values indicate higher avoidance of the affective stimulus (compared to the neutral stimulus) in MouseView.js compared to eye tracking, and negative values indicate higher avoidance of the affective stimulus in eye tracking. In the *top panels*, the *dashed line* indicates the average, and the *shaded area* the 95% confidence interval (based on between-participant pooled standard error of the mean, computed through Satterthwaite approximation). In the *bottom row*, Bayes factors quantify evidence for the alternative hypothesis (gaze and mouse are different) or the null hypothesis (gaze and mouse result in similar dwell-time differences). A log(BF_10_) of 1.1 corresponds with a BF_10_ of 3 (evidence for alternative), whereas a log(BF_10_) of – 1.1 corresponds with a BF_01_ of 3 (evidence for null)

We directly compared gaze and mouse dwell-time biases (Fig. 6, top row). We employed linear regression with an intercept, and with experiment sample membership (eye tracking or MouseView.js) as the sole predictor. This is analogous to an independent-samples*t* test, but has the benefit of allowing for a prior-free Bayes factor computation; using the Bayesian Information Criterion (BIC) from the regression, and from a null model with only an intercept (Wagenmakers, 2007). The log(BF_10_) is plotted in Fig. 6 (bottom row) and could be considered evidence for a difference between gaze and mouse from about 1.1 (corresponds with BF_10_ = 3), or evidence for the lack of a difference from – 1.1 (corresponds with BF_10_ = 1/3).

These results indicate that the initial approach of affective stimuli was stronger in gaze data, and that disgust avoidance in later stimulus repetitions is stronger in mouse data. However, there was no difference between eye tracking and MouseView.js for the majority of time in trials. This suggests that, after the first 1–1.5 s, MouseView.js is a good approximation of eye tracking in preferential looking tasks.

#### Relation to self-report

**Fig. 7:**
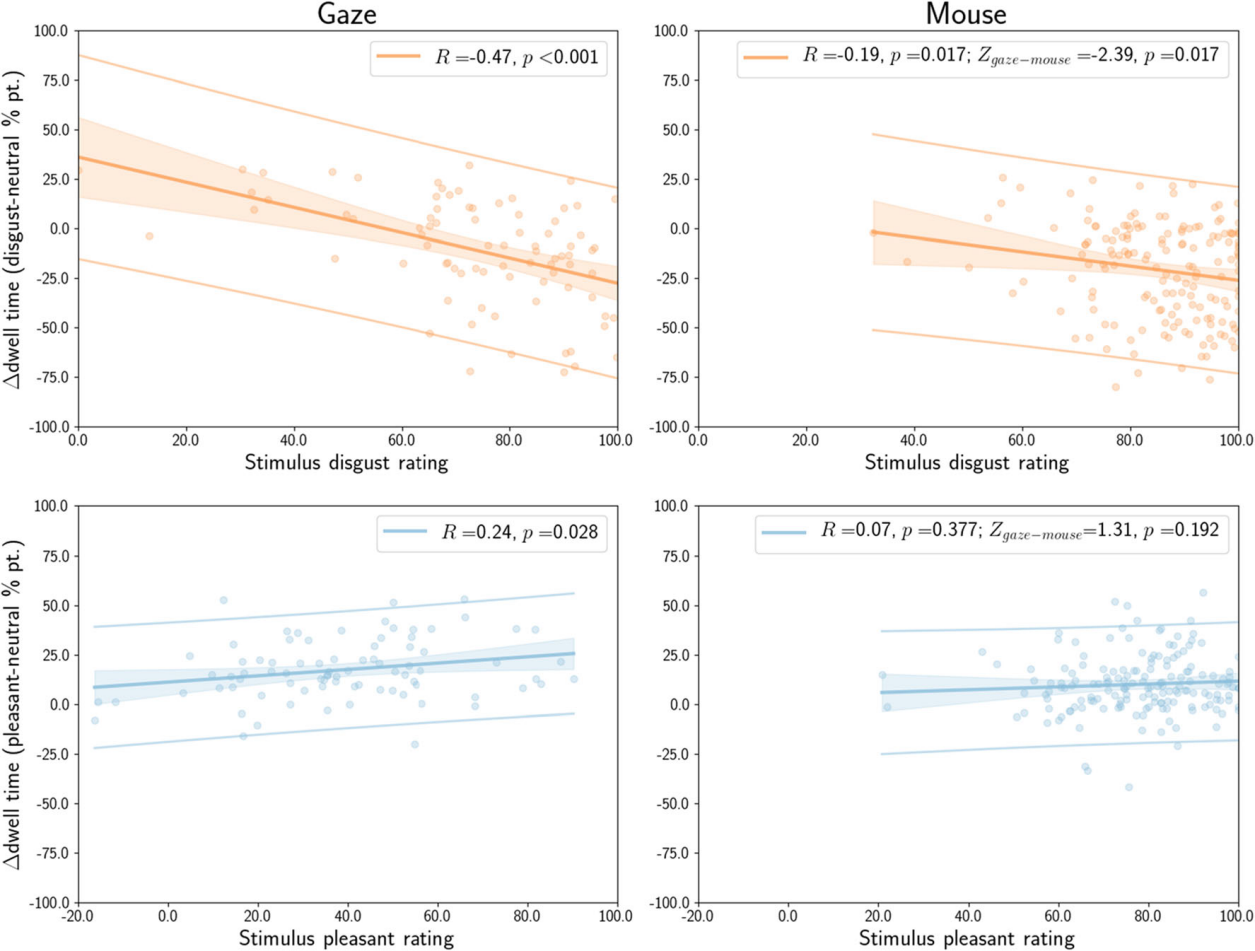
Correlation between the average self-reported disgust (*top row*) or pleasantness rating (*bottom row*) and the affective-neutral difference in dwell time. Ratings were averaged across all stimuli within each condition, and dwell times across all stimuli and stimulus presentations within each condition. The reported *Z* and uncorrected *p* values quantify the difference between dwell times obtained with eye tracking (*left column*) and MouseView.js (*right column*). *Solid lines* indicate the linear regression line, and the *shaded area* the error of the estimate

Averaged across all stimuli and stimulus repetitions, the average difference between dwell time for disgust and neutral items correlated with the average disgust rating for stimuli (Fig. 7, top row). This was true for gaze (*R* = – 0.47, *p* < 0.001) and for MouseView.js dwell (*R* = – 0.19, *p* = 0.017), although this correlation was significantly lower for mouse compared to gaze (*Z* = – 2.39, *p* = 0.017).

The average difference between dwell time for pleasant compared to neutral images was correlated with average pleasantness ratings (Fig. 7, bottom row) for gaze (*R* = 0.24, *p* = 0.028), but not for MouseView.js dwell times (*R* = 0.07, *p* = 0.377); although there was no significant difference between the two correlations (*Z* = 1.31, *p* = 0.192).

We also analysed whether self-reported stimulus disgust and pleasantness ratings impacted dwell times by employing linear mixed models. Here, we predicted gaze and mouse dwell time using a model with main factors condition (levels: disgust and pleasant), stimulus rating, and presentation number, and their interactions; and with participant number as random effect. These models showed highly similar outcomes for gaze (Table 4) and MouseView.js data (Table 5). Results indicated that participants showed more approach to pleasant stimuli compared to disgusting ones, less approach to stimuli with higher ratings (likely driven by disgust stimulus ratings), and increasingly less approach with presentation number. In addition, ratings had opposite effects between conditions (likely driven by higher avoidance for disgust, and no change or higher approach with higher pleasantness ratings), and there was also an interaction effect of condition and presentation number (likely driven by an increased tendency of avoidance of disgust stimuli at increasing presentations).

**Table 4:** Outcomes of a linear mixed model of gaze dwell time difference (affective - neutral) with participant number as random effect, and as fixed effects condition (disgust or pleasant), self-reported stimulus rating, presentation number, and their interactions

	*β*	*β 95% CI*		*t*	*p*
Intercept	– 0.15	– 0.23	– 0.08	– 3.98	<0.001
condition (reference: pleasant)	0.50	0.42	0.58	13.01	<0.001
stimulus rating	– 0.33	– 0.40	– 0.26	– 9.32	<0.001
presentation nr	– 0.08	– 0.13	– 0.03	– 2.94	0.004
condition*rating	0.41	0.32	0.49	9.65	<0.001
condition*presentation	0.14	0.07	0.21	3.79	<0.001
rating*presentation	– 0.02	– 0.08	0.04	– 0.77	0.445
condition*rating*presentation	0.04	– 0.03	0.11	1.05	0.299

**Table 5:** Outcomes of a linear mixed model of MouseView.js dwell time difference (affective - neutral) with participant number as random effect, and as fixed effects condition (disgust or pleasant), self-reported stimulus rating, presentation number, and their interactions

	*β*	*β 95% CI*		*t*	*p*
Intercept	– 0.36	– 0.41	– 0.32	– 14.37	< 0.001
condition (reference: pleasant)	0.76	0.72	0.80	34.64	< 0.001
stimulus rating	– 0.10	– 0.13	– 0.07	– 5.93	< 0.001
presentation nr	– 0.22	– 0.25	– 0.19	– 14.49	< 0.001
condition*rating	0.15	0.10	0.19	6.14	< 0.001
condition*presentation	0.31	0.27	0.35	14.19	< 0.001
rating*presentation	– 0.01	– 0.04	0.02	– 0.58	0.561
condition*rating*presentation	0.04	0.00	0.08	1.76	0.081

In sum, these data show that eye tracking and MouseView.js resulted in qualitatively similar patterns of correlation between disgust avoidance and self-report, and quantitatively similar patterns for pleasantness approach and self-report. However, as a general pattern, the concordance between self-report and dwell was somewhat smaller for MouseView.js compared to eye tracking. MouseView.js participants used less of the available rating range than eye-tracking participants, potentially because we used a narrower scale in the MouseView.js design that lacked intermediate scale labels (e.g. “slightly”, “moderate”, etc). This could have impacted the reported correlations.

#### MouseView.js and gaze scanpath similarity

Previous reports have already established a good overlap between mouse-aperture viewing, gaze, and visual saliency (Gomez et al., 2017; Jiang et al., 2015). Here, we aimed to replicate these findings by comparing traditional heatmaps, but also by comparing scanpaths between eye-tracking and MouseView.js experiments.

In eye-tracking research, heatmaps usually quantify the location and duration of gaze fixations, because these moments of relative stability of the eye are when most active vision occurs. In MouseView.js, because the fovea-like aperture is mouse-guided, the concept of fixations as moments of stability between ballistic saccades does not hold up. To be able to directly compare gaze and mouse data, we resampled each to 30 Hz, resulting in 300 samples per trial. In addition, we scaled all viewports (which vary in size between participants) so that display coordinates were a minimum of 0 and a maximum of 1. Data was also flipped so that the affective stimulus appeared to the left of the neutral stimulus.

**Fig. 8:**
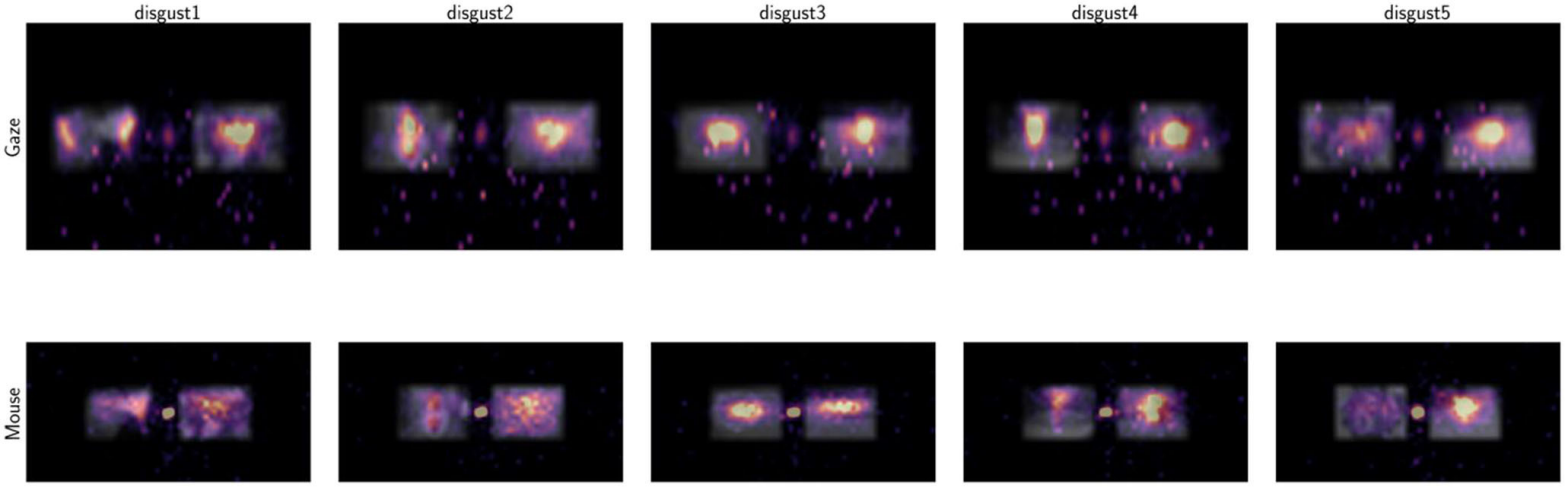
Heatmaps (two-dimensional histogram of resampled (*x*,*y*) coordinates) for all five disgust stimuli. The *top row* quantifies samples obtained from an eye-tracking experiment and the *bottom row* from a MouseView.js experiment. *Brighter colours* indicate more observations falling within that area. Note that, in reality, stimulus position was pseudo-random, but that samples were flipped where necessary so that the affective stimulus appeared on the left. Stimulus images are strongly blurred to obscure their content, as their usage license prevents publication

**Fig. 9:**
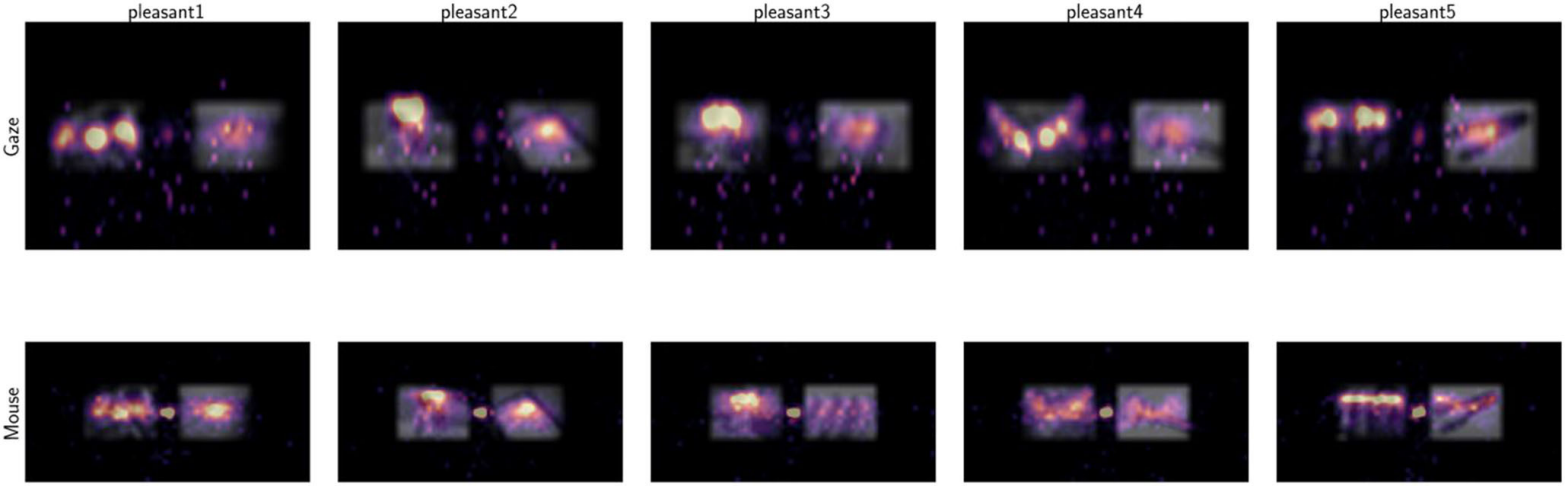
Heatmaps (two-dimensional histogram of resampled (*x*,*y*) coordinates) for all five pleasant stimuli. The *top row* quantifies samples obtained from an eye-tracking experiment and the *bottom row* from a MouseView.js experiment. *Brighter colours* indicate more observations falling within that area. Note that, in reality, stimulus position was pseudo-random, but that samples were flipped where necessary so that the affective stimulus appeared on the left. Stimulus images are strongly blurred to obscure their content, as their usage license prevents publication

Heatmaps were then constructed as two-dimensional histograms (Fig. 8 for disgust, and Fig. 9 for pleasant stimuli; see the Supplementary Material for larger versions). They illustrate that while some differences exist (likely due to subtle differences in experiment design), gaze and mouse patterns are qualitatively similar. Specifically, the spatial distribution across stimuli of the hottest areas (longest and/or most frequent dwell) aligned between gaze and mouse. This is apparent in two aspects: the global affective-neutral distribution, and the more local patterns within each image. Subtle differences exist too, most notably in the prominent central hotspot in mouse data, and the smearing of hot areas around specific points (particularly apparent in pleasant stimuli 1 and 5). Both were likely the result of mouse movements being slower than eye movements, first to move away from the location of the fixation cross that preceded stimulus presentation (also apparent in Fig. 5), and then in movement between points of interest.

**Fig. 10:**
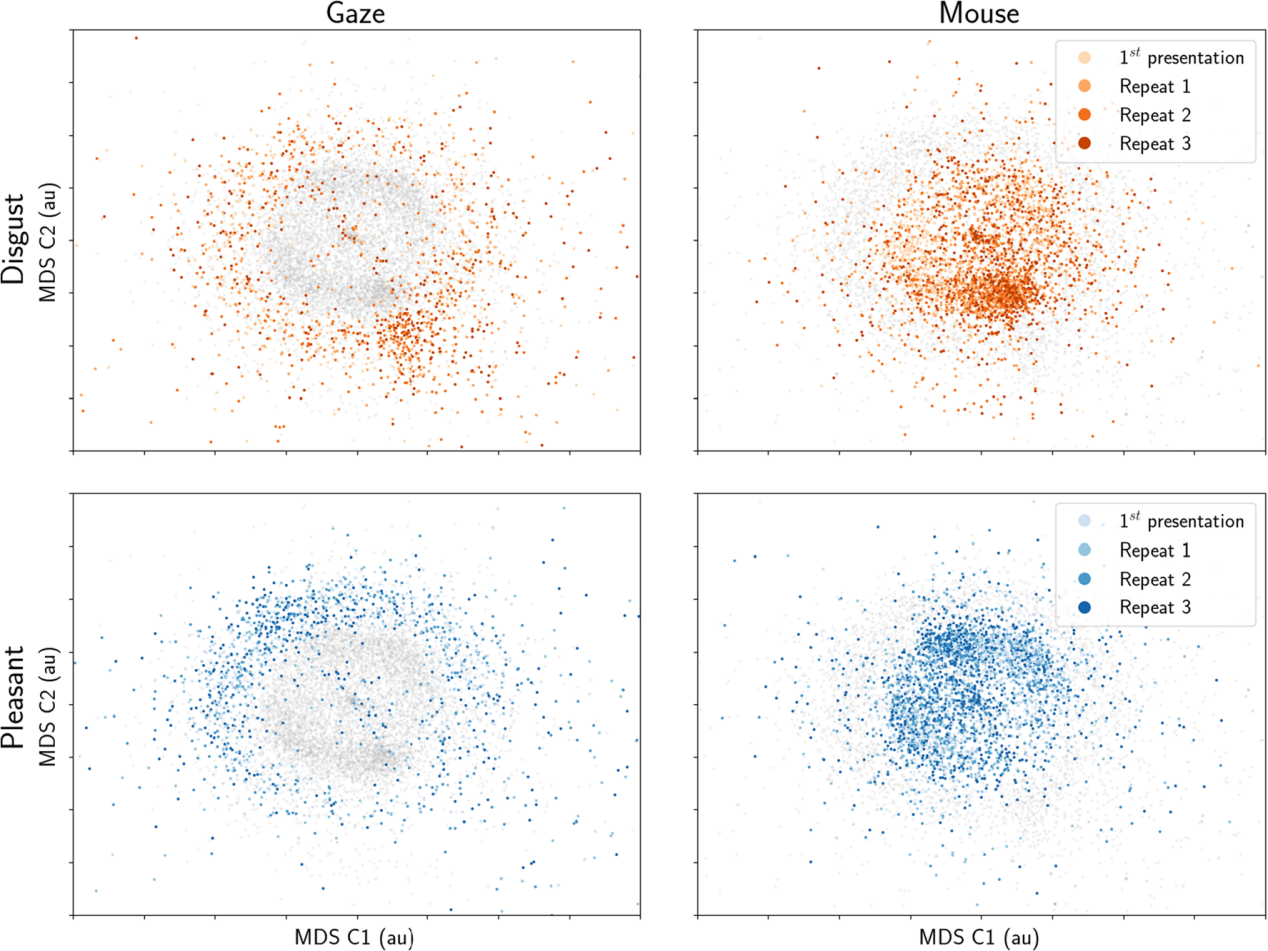
Three-dimensional scanpaths (horizontal and vertical coordinate, and time) reduced into two dimensions using multi-dimensional scaling (MDS). Each *dot* represents a single scanpath (i.e. a single trial). The *top row* shows scanpaths for disgust and neutral stimuli and the *bottom row* from the pleasant and neutral stimuli. The *left column* shows gaze (from eye tracking) scanpaths in colour and mouse (from MouseView.js) in *grey*; whereas the right column shows the opposite

**Fig. 11:**
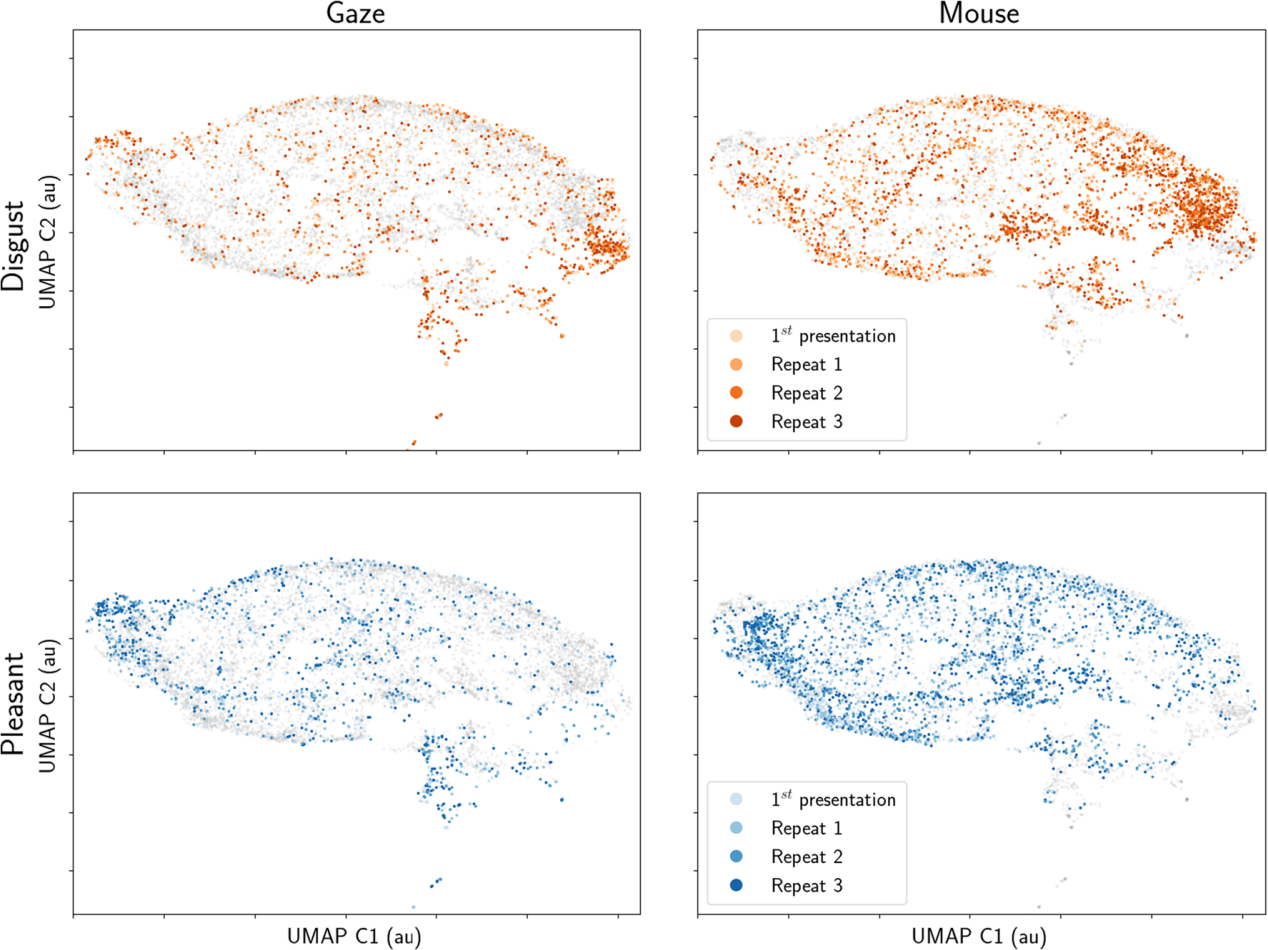
Three-dimensional scanpaths (horizontal and vertical coordinate, and time) reduced into two dimensions using uniform manifold approximation and projection (UMAP). Each *dot* represents a single scanpath (i.e. a single trial). The *top row* shows scanpaths for disgust and neutral stimuli and the *bottom row* from the pleasant and neutral stimuli. The *left column* shows gaze (from eye tracking) scanpaths in colour and mouse (from MouseView.js) in *grey*; whereas the *right column* shows the opposite

Where heatmaps are two-dimensional representations of gaze patterns, scanpaths also take into account the order of fixations, and sometimes their dwell duration. Hence, scanpaths are three-dimensional representations of gaze patterns. Traditionally, gaze fixations are extracted, after which their spatiotemporal patterns can be analyses (Cristino et al., 2010). However, as outlined before, mouse movements do not lend themselves well to fixation detection. Instead, to be able to directly compare gaze and mouse scanpaths, we constructed a single vector for each trial with 300 horizontal and 300 vertical coordinates from the resampled data (see above). Across all conditions, stimuli, and repetitions, this resulted in a combined matrix of 9852 rows (9920 trials; 9852 after excluding missing data) by 600 coordinates (300 horizontal and 300 vertical coordinates). This was then reduced into two dimensions (9852 x 2) using multi-dimensional scaling (MDS, Fig. 10)(Kruskal, 1964a, 1964b) or uniform manifold approximation and projection (UMAP, Fig. 11)(McInnes et al., 2018), so that scanpaths of all participants and trials could be fit into a single two-dimensional plot. This approach is similar to that taken by others to compare line path drawings to each other (Ang et al., 2018).

It was apparent, for both gaze and mouse data, that scanpaths for disgust and pleasant stimuli generally inhabited opposite ends of the reduced space, and that this is particularly true for later repetitions. This was expected, given the opposite avoidance and approach responses for disgust versus pleasant stimuli, and suggests that our scanpath reduction method was able to recognise dissociable patterns in the data.

Crucially, gaze and mouse data showed spatial differentiation in the MDS projection, and to a lesser extent in the UMAP projection. This means that gaze and mouse scanpaths shared features, but were qualitatively dissociable.

## Within-participants validation study

### Experiment

Our between-participant study showed that patterns of results from web-based data collection resembled those from an eye-tracking experiment. In this within-participant validation, we investigated how well MouseView.js approximates eye tracking within an individual. This is an important extension because it allowed us to directly compare gaze and mouse viewing behaviour in the same individuals.

The experiment and stimuli were identical to the MouseView.js experiment in the between-participants validation.

#### Procedure

Participants first completed demographic information, and provided ratings for each of the stimuli. For each methodology, the 40 trials were presented in two blocks, with participants alternating methodologies between blocks. They were randomly assigned to one of two orders: eye tracking first or MouseView.js first.

Eye tracking was conducted with a headrest, at a distance of 60 cm from the monitor (Dell P2219H, 22”, operating at a resolution of 1280 x 1024 at 60 Hz). Due to the COVID-19 pandemic, participants and experimenters wore masks, and abided by social-distancing rules. Because the eye tracker occasionally confused reflections on masks for those on the cornea, we introduced a policy of recalibrating prior to the second block. The experiment was run in OpenSesame (Mathôt et al., 2012), using the Expyriment (Krause & Lindemann, 2013) graphics back-end, and PyGaze (Dalmaijer et al., 2014) to control a GazePoint HD3 eye tracker (operating at 150 Hz).

The MouseView.js experiment was run via the Gorilla platform (Anwyl-Irvine, Dalmaijer, et al., 2020a; Anwyl-Irvine, Massonnié, et al., 2020b), on a separate computer from the eye-tracking experiment. Participants were not asked to use a headrest, so they were at a variable distance from the monitor (Dell P2219H, 22”, operating at a resolution of 1920 x 1080 at 60 Hz).

#### Participants

Participants were recruited among the student population at Whitman College (WA, USA). They were compensated $15 for the 35-min session.

Data were collected in March and April of 2021. Out of 50 participants, 70% self-identified as a woman, 24% as a man, and 6% as non-binary. Their age distribution spanned 18 to 23 years, with an average of 20.1 (median = 20) and a standard deviation of 1.22. Their racial/ethnic identity was 60% White, 22% Asian, 8% Latino/a, 4% Black, 4% multiracial; and 2% did not provide any.

One participant was excluded because the eye tracker could not be calibrated on them.

### Reliability

As before, we examined whether MouseView.js produced reliable results by computing the difference in dwell time between the affective and neutral stimulus in each trial. We then computed Cronbach’s coefficient α, and the split-half reliability as the average Spearman–Brown coefficient (ρ) over 100 different halfway-splits of all trials within each condition (disgust or pleasant).

The results are summarised in Table 6, and show that differences in dwell time between the affective and the neutral image in each trial were of good reliability. This was especially true for disgust stimuli, for both gaze (ρ = 0.92, α = 0.89) and mouse (ρ = 0.94, α = 0.91); and to a lesser extent for pleasant stimuli for both gaze (ρ = 0.73, α = 0.73) and mouse (ρ = 0.76, α = 0.74). These results illustrate that the reliability of dwell-difference measures is excellent (for disgust stimuli) to acceptable (for pleasant stimuli). Crucially, reliability is highly similar between eye-tracking and MouseView.js.

**Table 6:** The reliability of dwell-time differences between neutral and affective stimuli in 20 trials, estimated by the Spearman–Brownsplit-half reliability ρ (and the standard error of its mean across splits) over 100 random splits, and Cronbach’s coefficient α

Stimulus type	Eye tracking		Mouse view	
	ρ (se)	α	ρ (se)	α
Disgust	0.88 (2.64e-3)	0.85	0.94 (1.45e-3)	0.91
Pleasant	0.70 (6.68e-3)	0.68	0.76 (6.08e-3)	0.74

#### Behavioural consistency

As before, we computed the correlation in affective-neutral dwell-differences between different images (across different presentations), and between different presentations of the same images. This quantified the consistency in participants’ oculomotor avoidance (or approach) between different stimuli and repeated presentations of the same stimuli. Consistency partially reflects reliability, but is also impacted by the differences between stimulus images, and by stimulus repetition effects (Armstrong et al., 2020; Dalmaijer et al., 2021; Nord et al., 2021).

**Fig. 12:**
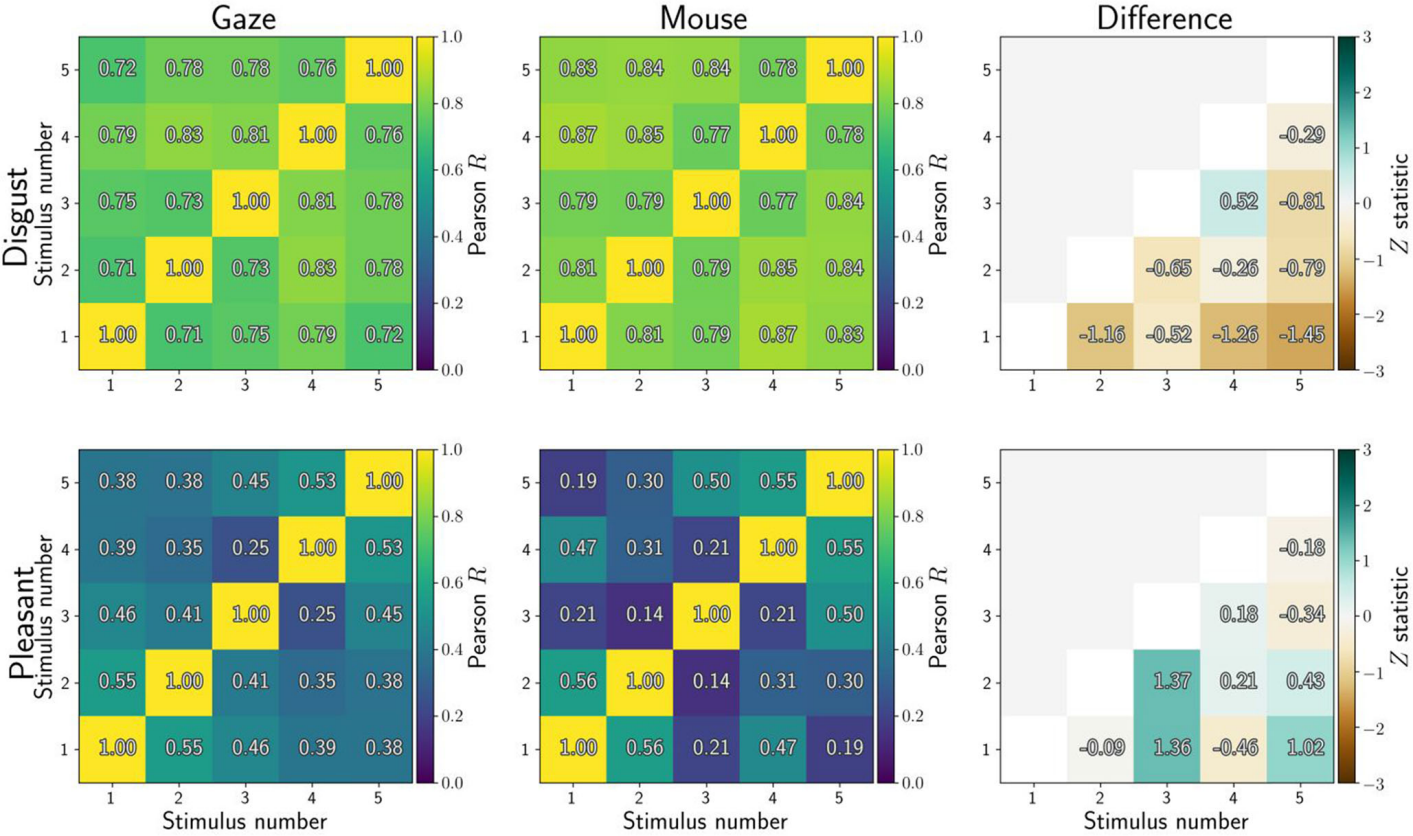
Correlations between affective-neutral dwell time differences for all stimuli (averaged across all four presentations). Higher correlations indicate that participants showed similar dwell time differences between stimuli. Included here are five disgust stimuli (*top row*) and five pleasant stimuli (*bottom row*). Dwell times were computed from eye tracking (*left column*) or MouseView.js (*middle column*), and the difference between them is reported in the *right column*

Gaze behaviour was highly consistent between disgust stimuli for both gaze (*R* = 0.72–0.84) and mouse (*R* = 0.77–0.86) dwell time, with no statistically significant differences between the two methods (Fig. 12). Gaze behaviour was less consistent between pleasant stimuli for both gaze (*R* = 0.23–0.0.54) and mouse (*R* = 0.16–0.58), again with no statistically significant differences between the two.

**Fig. 13:**
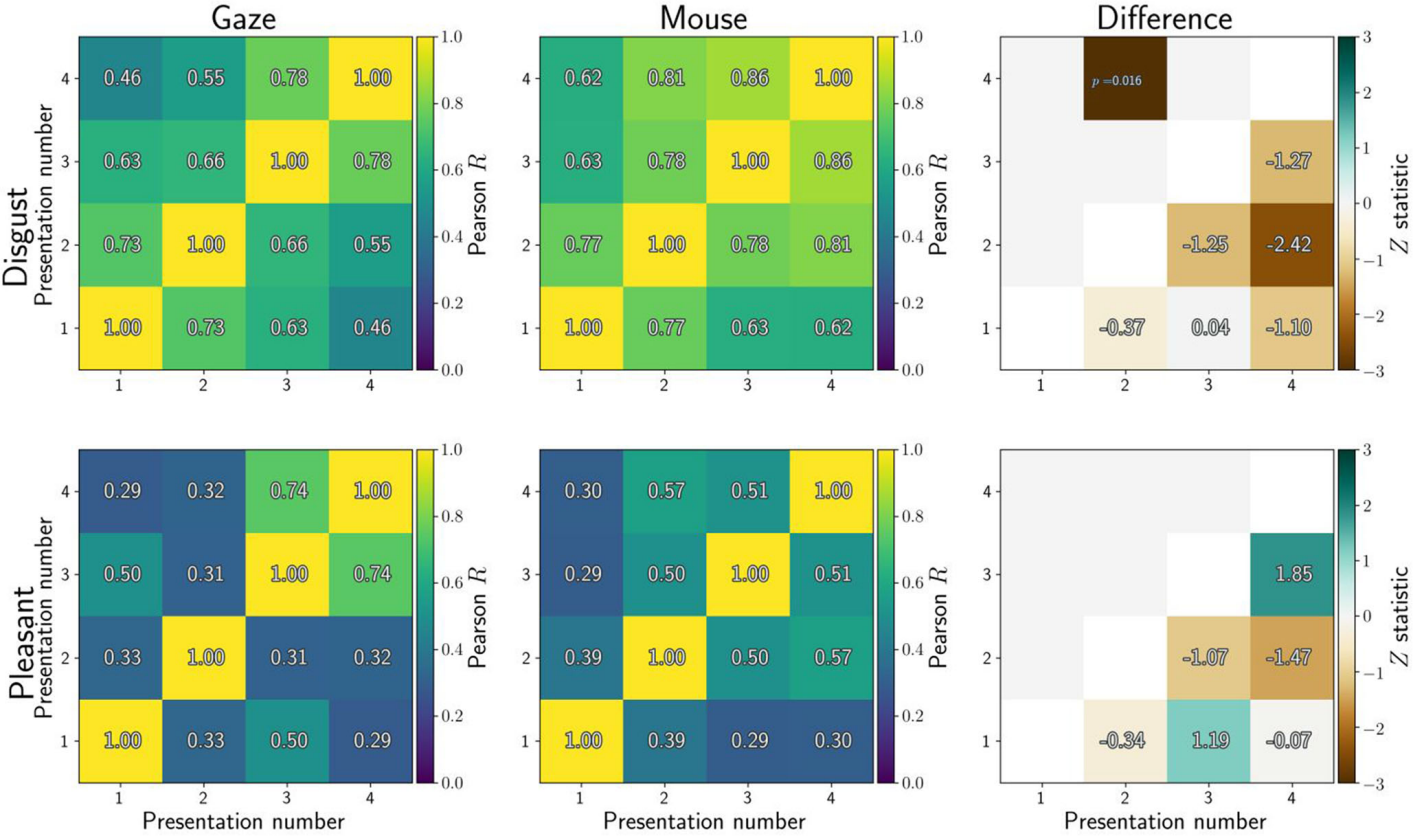
Correlations between affective-neutral dwell time differences for all stimuli (averaged across all four presentations). Higher correlations indicate that participants showed similar dwell time differences between stimuli. Included here are five disgust stimuli (*top row*) and five pleasant stimuli (*bottom row*). Dwell times were computed from eye tracking (*left column*) or MouseView.js (*middle column*), and the difference between them is reported in the *right column*

Gaze behaviour was also consistent between repeated presentations of the same disgust stimuli for both gaze (*R* = 0.45–0.79) and mouse (*R* = 0.64–0.87) dwell times Fig. 13, with only one (out of six) cell showing a statistically significant difference between gaze and mouse (second and fourth presentation, *R*_gaze_ = 0.54, *R*_mouse_ = 0.82, *Z* = – 2.66, *p* = 0.008). Consistency was lower for repetitions of the same pleasant stimuli for both gaze (*R* = 0.32–0.75) and mouse (*R* = 0.32–0.58), with no statistically significant differences between them.

These results indicate that participants’ gaze behaviour between different stimuli and repetitions was consistent, particularly for disgust stimuli. Crucially, behavioural consistency was not different between eye tracking and MouseView.js.

### Validity

In this validation study, we could directly compare eye tracking and MouseView.js within the same individuals. We did so by computing the difference in approach or avoidance between the two methods, to quantify whether and when there were offsets between the methods. In addition, we estimated the relationship between gaze and mouse dwell time, to establish whether the methods were similar even if there were any systematic offsets.

#### Approach or avoidance in gaze and mouse dwell times

**Fig. 14:**
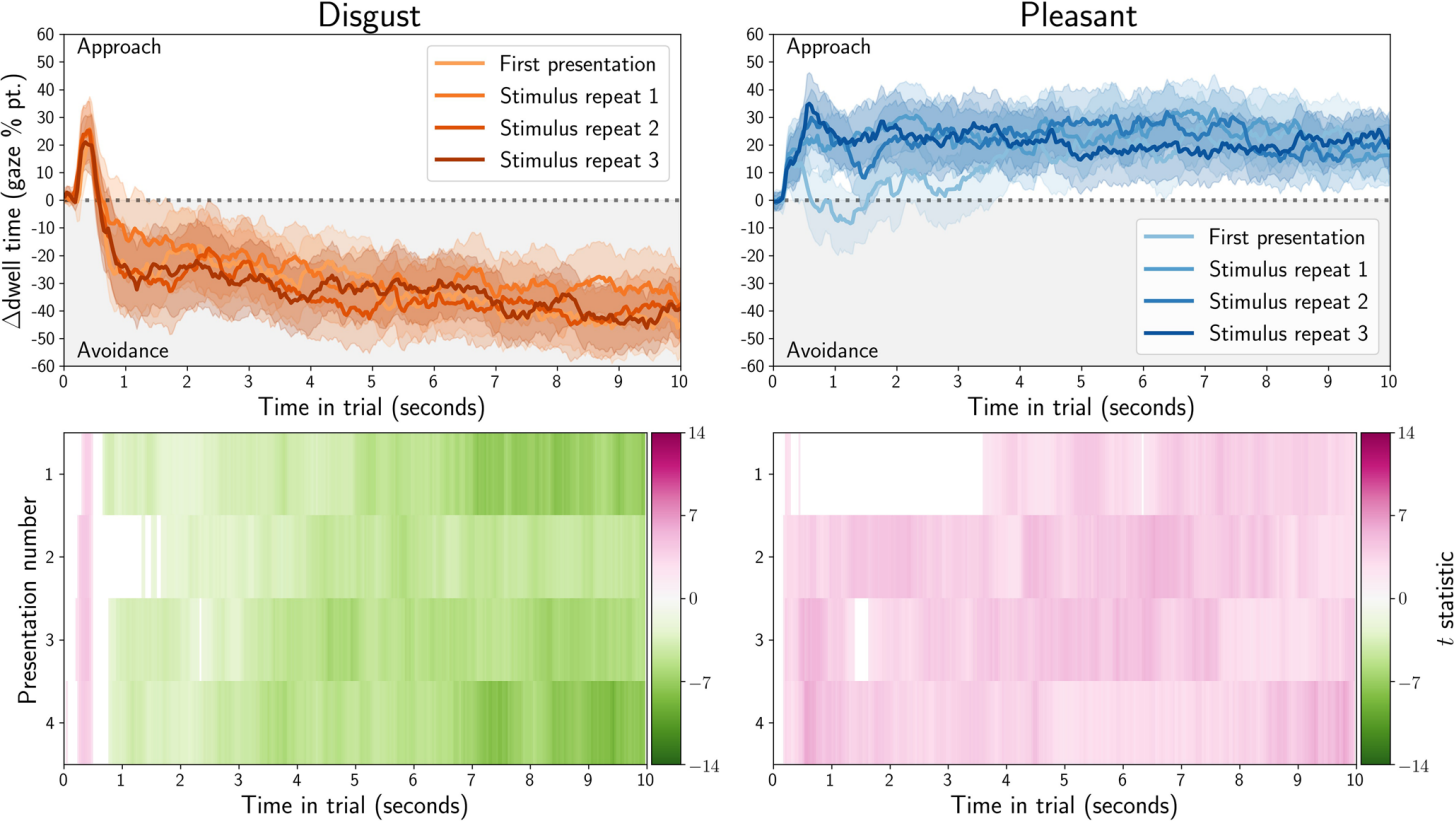
Gaze dwell time difference (in percentage points) between affective and neutral stimuli as obtained in an eye-tracking task. *Positive values* indicate participants spent more time looking at the affective (disgust or pleasant) stimulus than the neutral stimulus and *negative values* indicate the opposite. In the *top row*, *solid lines* indicate averages, and shading the within-participant 95% confidence interval. The *bottom row* shows *t* values from one-sample*t* tests of the dwell-difference compared to 0, for those tests where *p* < 0.05 (uncorrected). Positive *t* values (*pink*) indicate higher dwell time for the affective stimulus (approach), and negative *t* values (*green*) indicate higher dwell time for the neutral stimulus (avoidance)

**Fig. 15.**
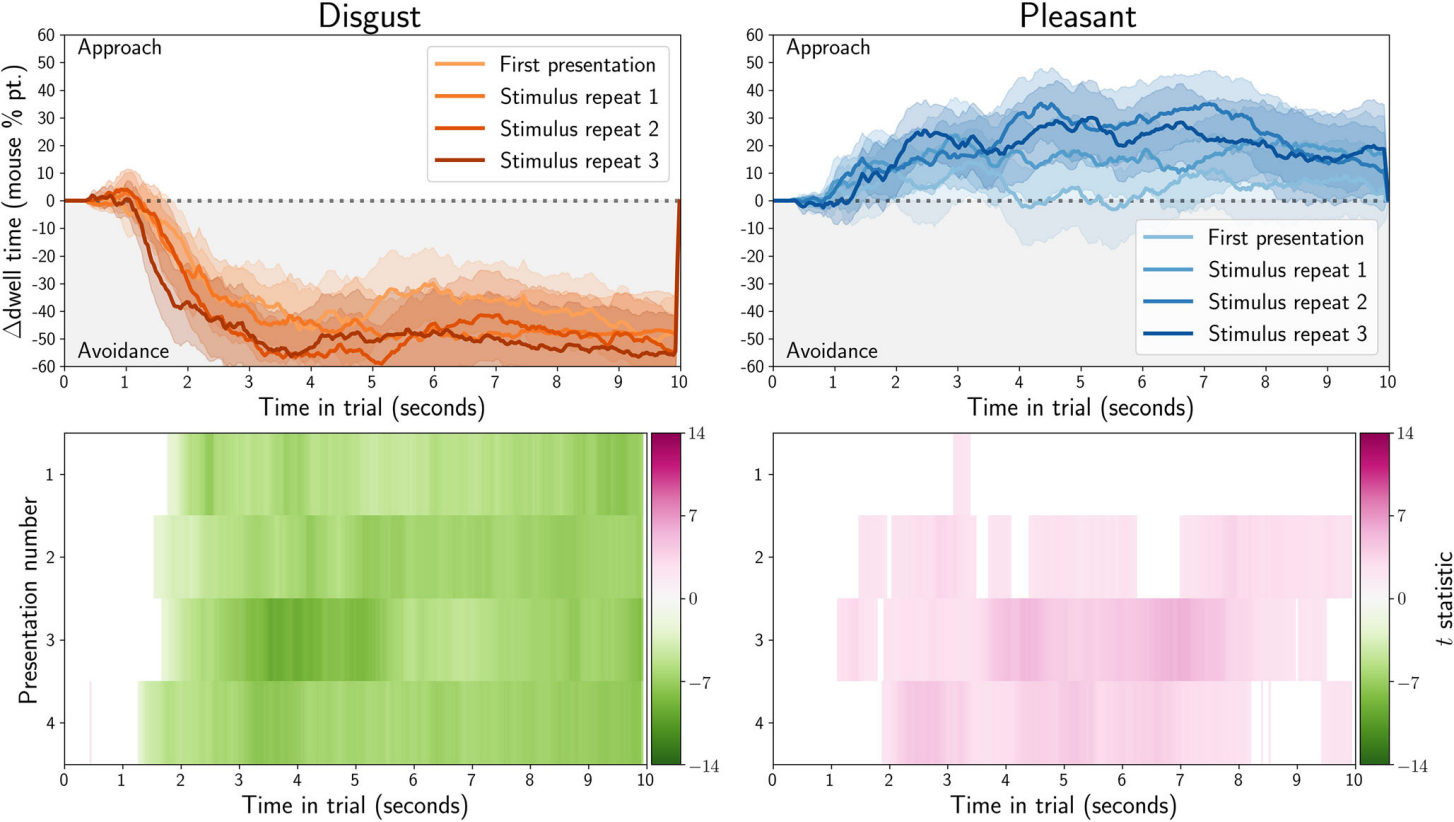
Mouse dwell time difference (in percentage points) between affective and neutral stimuli as obtained in a web-based MouseView.js task. *Positive values* indicate participants spent more time looking at the affective (disgust or pleasant) stimulus than the neutral stimulus and *negative values* indicate the opposite. In the *top row*, *solid lines* indicate averages, and shading the within-participant 95% confidence interval. The sharp return to 0 at the end of the trial duration is an artefact of mouse recording cutting out slightly too early. The *bottom row* shows *t* values from one-sample*t* tests of the dwell-difference compared to 0, for those tests where *p* < 0.05 (uncorrected). Positive *t* values (*pink*) indicate higher dwell time for the affective stimulus (approach), and negative *t* values (*green*) indicate higher dwell time for the neutral stimulus (avoidance)

As before, dwell time was computed as the total duration of gaze (Fig. 14) or mouse (Fig. 15) samples for which the coordinate was within an image. The difference in dwell time for affective and neutral stimuli was computed between each of the stimulus pairs, and then averaged over all stimuli within each condition (disgust or pleasant). The dwell time difference thus quantified the extent to which participants preferred the affective over the neutral stimulus, i.e. whether they showed approach or avoidance of the affective stimulus.

Dwell differences are plotted in for eye tracking and for MouseView.js. In addition, one sample *t* tests of the difference were computed, and plotted in the bottom rows. These quantify the magnitude of the difference, and whether it is statistically significantly different from zero.

As in the between-participants study, participants showed sustained bias towards pleasant stimuli and away from disgust stimuli. As before, the exception to this was an initial, short-lived bias towards disgust stimuli that was apparent in the gaze but not the mouse data.

#### Direct comparisons of eye tracking and MouseView.js dwell times

**Fig. 16:**
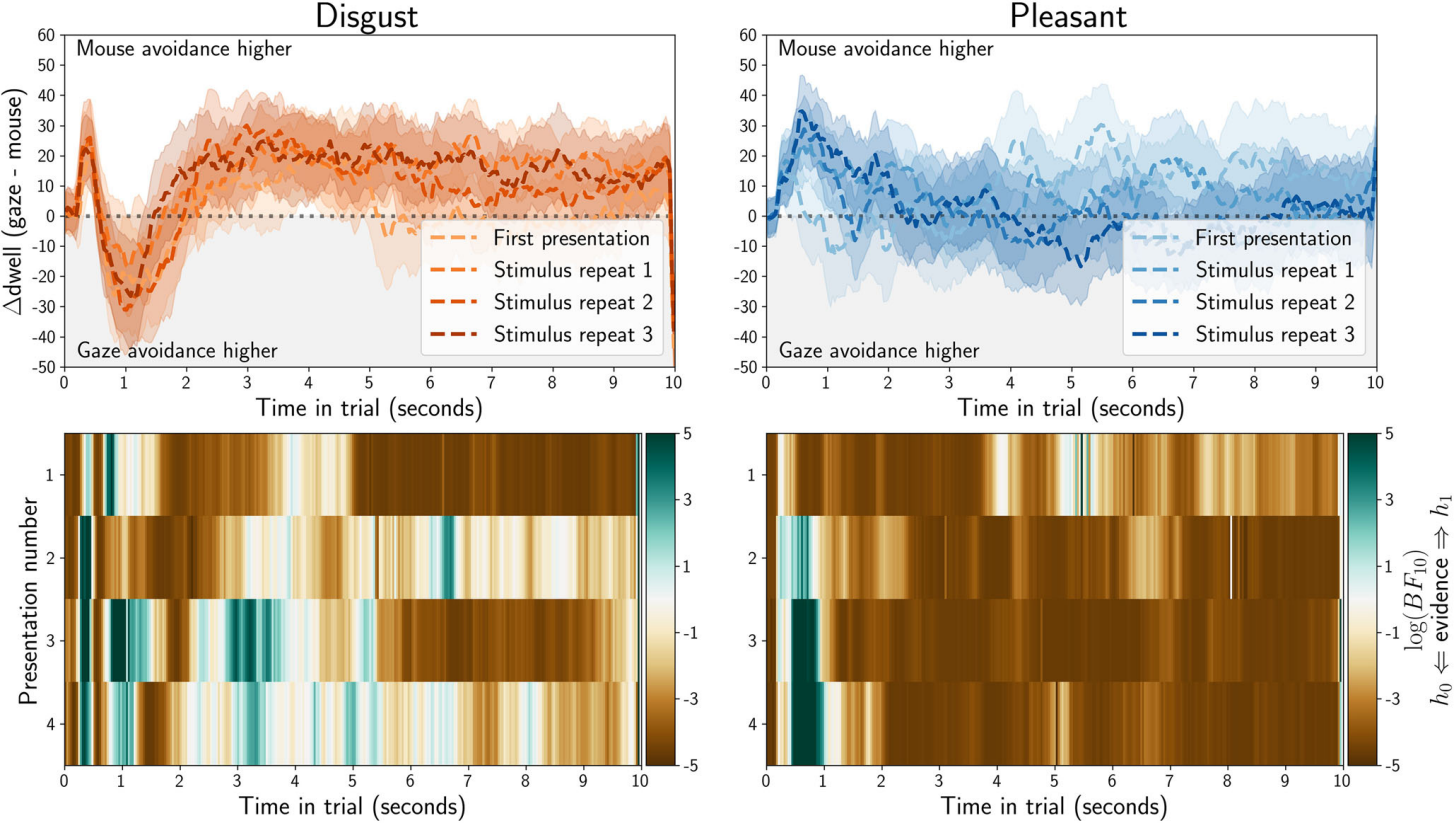
Quantification of the difference between gaze dwell time differences (eye tracking, Fig. 14) and mouse dwell time differences (MouseView.js, Fig. 15). *Positive values* indicate higher avoidance of the affective stimulus (compared to the neutral stimulus) in MouseView.js compared to eye tracking and *negative values* indicate higher avoidance of the affective stimulus in eye tracking. In the *top panels*, *dashed lines* indicate the average and the *shaded area* the 95% within-participant confidence interval. In the *bottom panels*, Bayes factors quantify evidence for the alternative hypothesis (gaze and mouse are different) or the null hypothesis (gaze and mouse are not different); each quantified as a linear mixed model with participant as random effect, and the alternative model with method (gaze/mouse) as fixed effect. A log(BF_10_) of 1.1 corresponds with a BF_10_ of 3 (evidence for the alternative), whereas a log(BF_10_) of – 1.1 corresponds with a BF_01_ of 3 (evidence for the null)

We directly compared gaze and mouse dwell time (Fig. 16, top row), and used linear mixed models to statistically test differences between the two methods. Specifically, for each time bin and each presentation, we computed the average difference between gaze and mouse dwell-differences between affective and neutral stimuli (i.e. the difference in approach/avoidance between gaze and mouse). The alternative model of this dwell difference had method (gaze or mouse) as fixed effect and participant as random effect; whereas the null model comprised only an intercept and participant as random effect. We computed the Bayesian Information Criterion for each alternative and null model, and used this to compute Bayes factors (Wagenmakers, 2007). To improve the accessibility of the visualisation, we then computed the log of each Bayes factor (Fig. 16, bottom row). Here, positive values (blue) indicate evidence for the hypothesis that dwell time differences are different between eye-tracking and MouseView.js, and negative values (brown) indicate evidence for the hypotheses that dwell time differences are similar between the methods.

These results showed that initial approach of the affective stimulus was stronger in eye tracking, but that overall there was evidence for eye tracking and MouseView.js producing similar dwell time differences between affective and neutral stimuli. This suggests that, after the first 1–1.5 s, MouseView.js is a good approximation of eye tracking in preferential looking tasks.

Because participants in this study did the same experiments with eye tracking and MouseView.js, we could compute the correlation between dwell time differences for both techniques. For each technique and time bin, we computed the difference in dwell time between affective and neutral stimuli, averaged across all stimuli and presentations. We then correlated the gaze and mouse dwell time differences averaged across time bins, and for each time bin.

**Fig. 17:**
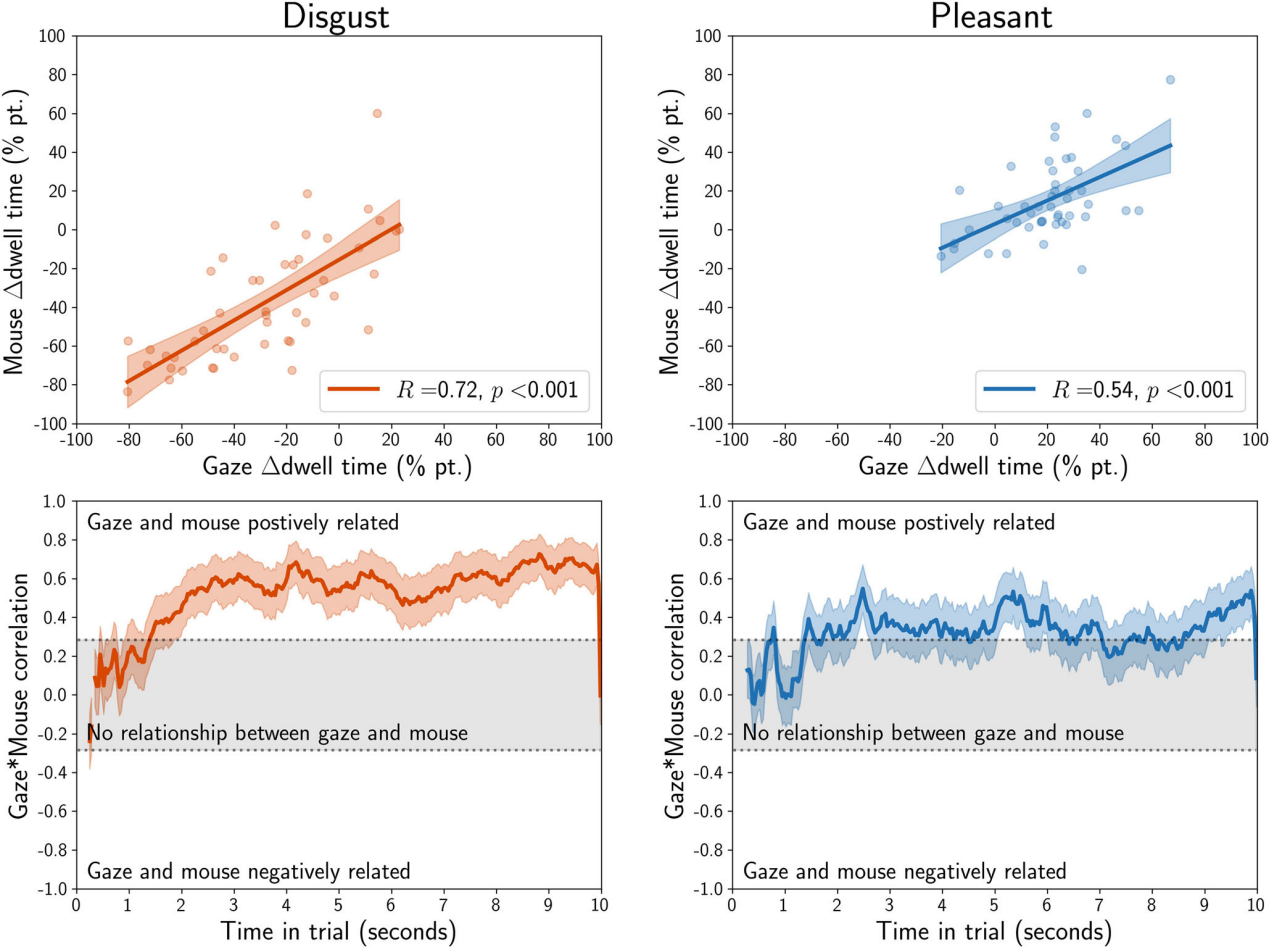
Quantification of the relationship between eye tracking and MouseView.js dwell time differences between disgust (*red*) or pleasant (*blue*) and neutral stimuli. The *top panels* show the regression line, with the error of the estimate shaded, and individuals plotted as *dots*. The *bottom panels* show the Pearson correlation between gaze and mouse per time bin (*solid line*) and its standard error (*shaded area*). Values that fall within the grey area are not statistically significant, with the *dotted lines* indicating the critical values for *R* where *p* = 0.05

Average gaze and mouse dwell time differences correlated strongly for both disgust (*R* = 0.72, *p* < 0.001) and pleasant (*R* = 0.54, *p* < 0.001) stimuli (Fig. 17, top row). For both stimulus types, the correlation between gaze and mouse was statistically significant from about 1.5 s into a trial, albeit more consistently so for disgust stimuli than for pleasant stimuli (Fig. 17, bottom row).

#### Relation to self-report

**Fig. 18:**
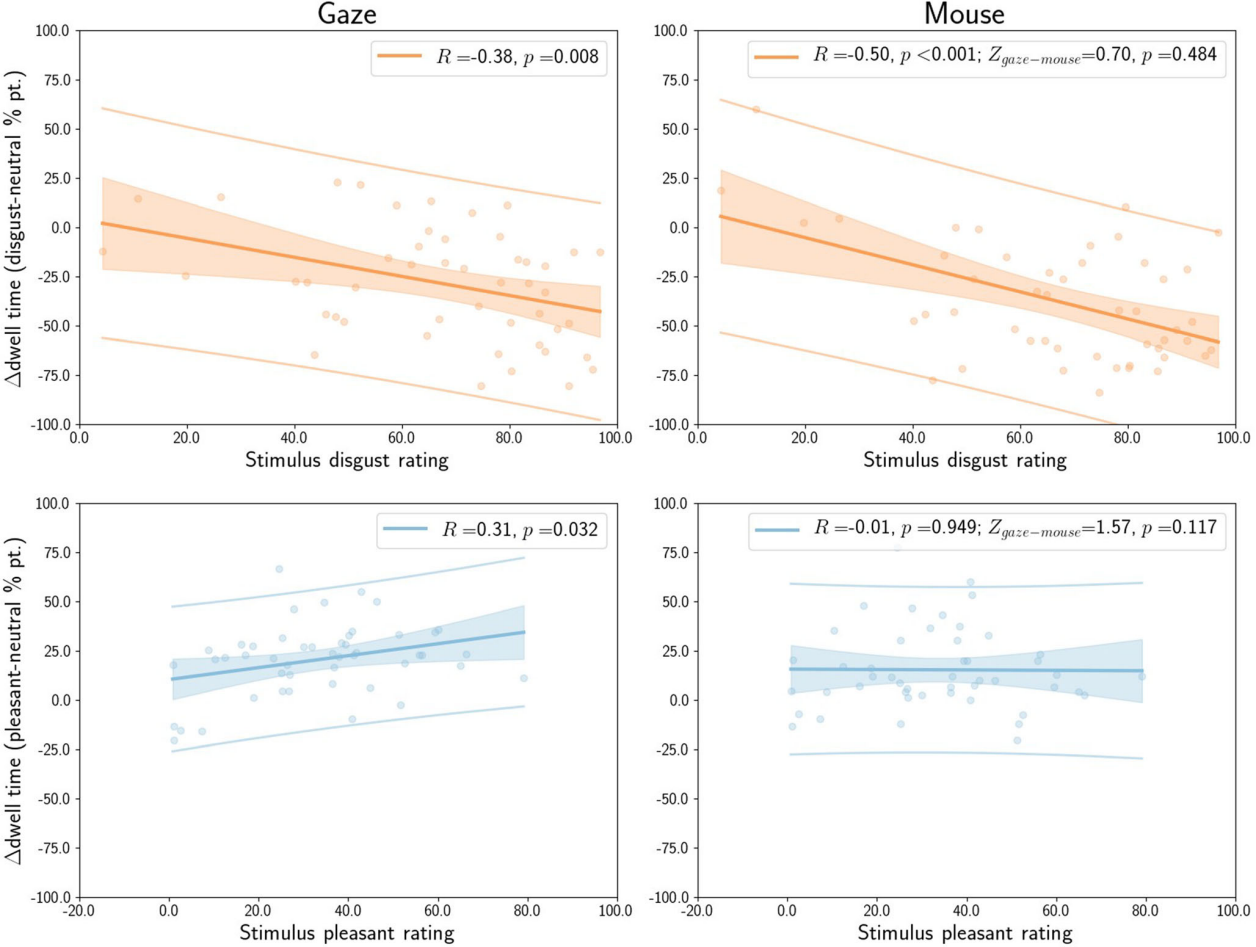
Correlation between the average self-reported disgust (*red*, *top row*) or pleasantness rating (*blue*, *bottom row*) and the affective-neutral difference in dwell time. Ratings were averaged across all stimuli within each condition, and dwell times across all stimuli and stimulus presentations within each condition. The reported *Z* and (uncorrected)*p* values quantify the difference between the correlations for eye tracking (*left column*) and MouseView.js (*right column*). *Solid lines* indicate the linear regression line and the *shaded area* the error of the estimate. *Dots* represent individual participants

Averaged across all stimuli and stimulus presentations, the average difference between dwell time for disgust and neutral stimuli correlated with the average disgust rating for stimuli (Fig. 18, top row). This was true for eye tracking (*R* = – 0.38, *p* = 0.008) and for MouseView.js (*R* = – 0.50, *p* < 0.001), and there was no statistically significant difference in the magnitude of these correlations (*Z* = 0.70, *p* = 0.484).

The average difference between dwell time for pleasant and neutral images was correlated with the average pleasantness rating (Fig. 18, bottom row) for eye tracking (*R* = 0.31, *p* = 0.032), but not for MouseView.js (*R* = – 0.01, *p* = 0.949); although there was no statistically significant difference in the magnitude of these correlations (*Z* = 1.57, *p* = 0.117).

These findings closely resemble those of the between-participants study (22).

### What drives dwell time differences

We analysed what factors related to the difference in dwell time between affective and neutral stimuli, using linear mixed models. We considered main effects of method (levels: gaze and mouse), condition (levels: disgust and pleasant), stimulus rating, and presentation number; their interactions; and participant number as random effect. The best-fitting model included condition and stimulus rating as fixed effects, their interaction, and participant number as random effect. It is summarised in Table 7.

**Table 7:** Outcomes of a linear mixed model of dwell time difference (affective-neutral) with participant number as random effect, and as fixed effects condition (disgust or pleasant), self-reported stimulus rating, and its interaction

Intercept	*β*	*β 95% CI*		*t*	*p*
	– 0.38	– 0.47	– 0.30	– 9.25	< 0.001
condition (reference: pleasant)	0.91	0.85	0.98	28.69	< 0.001
stimulus rating	– 0.23	– 0.28	– 0.18	– 9.49	< 0.001
condition*rating	0.24	0.18	0.31	7.52	< 0.001

The second-best fitting model was similar to the first, but it did include method (gaze or mouse) and its interactions. It showed a statistically significant main effect of method (β = – 0.15 [– 0.24, – 0.07], *t* = – 3.52, *p* = 0.001). However, the fact that this model did not fit the data as well (ΔBIC = 7.78) could be taken as evidence against a meaningful effect of method on dwell time differences.

## Discussion

We presented MouseView.js, a JavaScript tool that mimics human vision and eye movements with a mouse-locked aperture. We investigated its reliability and validity for preferential looking tasks in a replication of an eye-tracking study, in which participants were repeatedly presented with the same pairs of affective (disgust or pleasant) and neutral stimuli. We did so in a new sample of participants recruited for a web-based experiment, which we compared with existing gaze data. In addition, we ran a study in which participants were invited to the lab to complete both experiments, allowing for a direct comparison of gaze and mouse methods in the same individuals. We found the expected dwell differences in both gaze and mouse data, specifically sustained avoidance of disgust, and sustained approach of pleasant stimuli. Importantly, we found that MouseView.js was equally (disgust condition) or more (pleasant condition) reliable than eye tracking. We also found that MouseView.js was a valid alternative to eye tracking in preferential looking tasks for three main reasons: 1) There was evidence against a difference between results obtained from gaze and mouse methods (both between and within participants), 2) There was a high correlation between results obtained from gaze and mouse methods (within participants), and 3) Gaze and mouse dwell time differences were explained by the same best-fitting linear mixed models (between-participants), or were best explained by a linear mixed model that did not include mouse/gaze method as fixed effect (within-participants).

### Similarities between MouseView.js and eye tracking

We have replicated earlier work (Gomez et al., 2017; Jiang et al., 2015) by showing that gaze and mouse exploration heatmaps are qualitatively similar.

Preferential looking (sometimes referred to as “selective looking”) has a long history in developmental psychology (Hirsh-Pasek & Golinkoff, 1996; Teller, 1979), and has emerged as a popular tool in affective science (Armstrong & Olatunji, 2012; Mogg et al., 2000). Here, we replicated such a study, and show that MouseView.js dwell differences between affective and neutral stimuli quantitatively and qualitatively replicated the sustained oculomotor avoidance of disgusting and approach of pleasant stimuli reported in earlier work (Armstrong et al., 2020; Dalmaijer et al., 2021).

In addition, we show that differences in dwell time for affective compared to neutral stimuli (i.e. disgust avoidance or pleasant approach) correlate between MouseView.js and eye tracking, when these are measured in the same individuals. Notably, this correlation emerges only after about 1.5 s into a trial, suggesting that the overlap between mouse and gaze dwell time is driven by deliberate exploration of stimuli.

### Differences between MouseView.js and eye tracking

In the gaze data analysed here and in previous studies (Armstrong et al., 2020; Dalmaijer et al., 2021; Nord et al., 2021), disgust stimuli are subject to brief initial approach before sustained avoidance. MouseView.js data for the same stimuli showed a blunted and elongated initial approach in the first exposure to a disgust stimulus, and only sustained avoidance in all consecutive presentations. For pleasant and neutral stimulus pairs, initial approach of the pleasant stimulus was also more obvious for gaze compared to MouseView.js data, and followed by sustained approach for both methods thereafter. This pattern was obvious in dwell time differences for disgusting and neutral stimuli, and a likely driver of the qualitative differences between gaze and mouse scan paths.

Another difference between eye-tracking and MouseView.js methodology was the degree to which dwell time differences correlated with self-report in the between-participants study. For pairs of disgusting and neutral stimuli, gaze dwell difference correlated negatively with self-reported disgust. Mouse dwell also correlated negatively, but to a lesser extent. However, this difference was not replicated in the (in-lab)within-participant study, which did not show a statistically significant difference between mouse and gaze (and the difference was numerically in the opposite direction compared to the between-subjects study). Hence, the reduced correlation between dwell-based disgust avoidance and self-reported disgust could be a consequence of running the study via the Internet as opposed to in the lab. For pairs of pleasant and neutral stimuli, no such differences were apparent.

One reason for the initial gaze bias towards affective stimuli could be that eye movements are harder to suppress than mouse movements. This would make eye tracking more sensitive to the early and relatively automatic capture of attention by affective stimuli revealed by eye movements (Bradley et al., 2015; Mulckhuyse & Dalmaijer, 2016), and MouseView.js more sensitive to deliberate exploration.

### When to use MouseView.js to replace eye tracking

The results presented here suggest that MouseView.js approximates results from eye tracking when participants engage in voluntary exploration of stimuli, but that it failed to detect more reflexive capture of attention. As a consequence, MouseView.js would be a poor replacement for eye tracking in research on early automatic processes, such as oculomotor capture (Theeuwes et al., 1998) or the global effect (Van der Stigchel & de Vries, 2015).

Perhaps obviously, participants might not be able to produce saccade-like data with their mouse: it only moves as quick as the hand, and its position is logged at a considerably lower frequency than high-end eye trackers. It would thus be unwise to turn to MouseView.js for research on saccade dynamics, including trajectories (Van der Stigchel et al., 2006), inhibition or facilitation of return (Mills et al., 2015), and peak velocity (Muhammed et al., 2020).

MouseView.js is a good replacement for eye tracking in research on more deliberate behaviour, including preferential looking (used here), teleforaging (Manohar & Husain, 2013), and free viewing. In addition, MouseView.js can be a stand-in replacement for aperture-based research on reading (Rayner, 2014).

### Conclusion

MouseView.js is a new implementation of the decades-old method of forcing participants to move a clear aperture to visually explore an otherwise blurred stimulus display. We showed that this method is as reliable as eye tracking for preferential looking paradigms. In addition, we demonstrated qualitative if not always quantitative overlap between gaze and mouse dwell time and scanpaths. Our software has been made available as open-source JavaScript library, and implemented in web-based experiment builders Gorilla and PsychoPy, and scripting toolbox jsPsych.

## Supplementary Information

ESM 1 (PDF 7215 kb)

## References

[CR1] Ang Y-S, Manohar S, Plant O, Kienast A, Le Heron C, Muhammed K, Hu M, Husain M (2018). Dopamine modulates option generation for behavior. Current Biology.

[CR2] Anwyl-Irvine, A. L., Dalmaijer, E. S., Hodges, N., & Evershed, J. K. (2020a). Realistic precision and accuracy of online experiment platforms, web browsers, and devices. *Behavior Research Methods*. 10.3758/s13428-020-01501-5 10.3758/s13428-020-01501-5 PMC8367876 33140376

[CR3] Anwyl-Irvine AL, Massonnié J, Flitton A, Kirkham N, Evershed JK (2020). Gorilla in our midst: An online behavioral experiment builder. Behavior Research Methods.

[CR4] Armstrong T, Olatunji BO (2012). Eye tracking of attention in the affective disorders: A meta-analytic review and synthesis. Clinical Psychology Review.

[CR5] Armstrong, T., Stewart, J. G., Dalmaijer, E. S., Rowe, M., Danielson, S., Engel, M., Bailey, B., & Morris, M. (2020). I’ve seen enough! Prolonged and repeated exposure to disgusting stimuli increases oculomotor avoidance. *Emotion*. 10.1037/emo0000919 10.1037/emo0000919 33252938

[CR6] Bethlehem RAI, Dumoulin SO, Dalmaijer ES, Smit M, Berendschot TTJM, Nijboer TCW, Van der Stigchel S (2014). Decreased fixation stability of the preferred retinal location in juvenile macular degeneration. PLoS ONE.

[CR7] Blackwell, A. F., Jansen, A. R., & Marriott, K. (2000). Restricted Focus Viewer: A tool for tracking visual attention. *Proceedings of the First International Conference on Theory and Application of Diagrams*, 162–177.

[CR8] Bradley MM, Costa VD, Lang PJ (2015). Selective looking at natural scenes: Hedonic content and gender. International Journal of Psychophysiology.

[CR9] Bridges D, Pitiot A, MacAskill MR, Peirce JW (2020). The timing mega-study: comparing a range of experiment generators, both lab-based and online. PeerJ.

[CR10] Cristino F, Mathôt S, Theeuwes J, Gilchrist ID (2010). ScanMatch: A novel method for comparing fixation sequences. Behavior Research Methods.

[CR11] Dalmaijer ES, Mathôt S, Van der Stigchel S (2014). PyGaze: An open-source, cross-platform toolbox for minimal-effort programming of eyetracking experiments. Behavior Research Methods.

[CR12] Dalmaijer, E. S., Lee, A., Leiter, R., Brown, Z., & Armstrong, T. (2021). Forever yuck: Oculomotor avoidance of disgusting stimuli resists habituation. *Journal of Experimental Psychology: General*. 10.1037/xge0001006 10.1037/xge0001006 PMC7613016 33475396

[CR13] de Leeuw JR (2015). jsPsych: A JavaScript library for creating behavioral experiments in a Web browser. Behavior Research Methods.

[CR14] Deng J, Krause J, Fei-Fei L (2013). Fine-Grained Crowdsourcing for Fine-Grained Recognition. IEEE Conference on Computer Vision and Pattern Recognition.

[CR15] Gomez SR, Jianu R, Cabeen R, Guo H, Laidlaw DH (2017). Fauxvea: Crowdsourcing gaze location estimates for visualization analysis tasks. IEEE Transactions on Visualization and Computer Graphics.

[CR16] Gómez-Poveda J, Gaudioso E (2016). Evaluation of temporal stability of eye tracking algorithms using webcams. Expert Systems with Applications.

[CR17] Gosselin F, Schyns PG (2001). Bubbles: a technique to reveal the use of information in recognition tasks. Vision Research.

[CR18] Hirsh-Pasek K, Golinkoff RM, McDaniel D, McKee C, Cairns HS (1996). The intermodal preferential looking paradigm: A window onto emerging language comprehension. Language, speech, and communication. Methods for assessing children’s syntax.

[CR19] Holmqvist, K., Nystrom, M., Andersson, R., Dewhurst, R., Jarodzka, H., & Van de Weijer, J. (2015). *Eye tracking: a comprehensive guide to methods and measures* (First published in paperback). Oxford University Press.

[CR20] Itti L, Koch C, Niebur E (1998). A model of saliency-based visual attention for rapid scene analysis. IEEE Transactions on Pattern Analysis and Machine Intelligence.

[CR21] Jansen AR, Blackwell AF, Marriott K (2003). A tool for tracking visual attention: The Restricted Focus Viewer. Behavior Research Methods, Instruments, & Computers.

[CR22] Jiang, M., Huang, S., Duan, J., & Zhao, Q. (2015). SALICON: Saliency in Context. *Proceedings of the IEEE Conference on Computer Vision and Pattern Recognition*, 1072–1080.

[CR23] Kazemi V, Sullivan J (2014). One millisecond face alignment with an ensemble of regression trees. IEEE Conference on Computer Vision and Pattern Recognition.

[CR24] Kim, N. W., Bylinskii, Z., Borkin, M. A., Oliva, A., Gajos, K. Z., & Pfister, H. (2015). A Crowdsourced Alternative to Eye-tracking for Visualization Understanding. *Proceedings of the 33rd Annual ACM Conference Extended Abstracts on Human Factors in Computing Systems*, 1349–1354. 10.1145/2702613.2732934

[CR25] Kim NW, Bylinskii Z, Borkin MA, Gajos KZ, Oliva A, Durand F, Pfister H (2017). BubbleView: An interface for crowdsourcing image importance maps and tracking visual attention. ACM Transactions on Computer-Human Interaction.

[CR26] Krafka, K., Khosla, A., Kellnhofer, P., Kannan, H., Bhandarkar, S., Matusik, W., & Torralba, A. (2016). Eye tracking for everyone. *IEEE Conference on Computer Vision and Pattern Recognition (CVPR)*.

[CR27] Krause, F., & Lindemann, O. (2013). Expyriment: A Python library for cognitive and neuroscientific experiments. *Behavior Research Methods*. 10.3758/s13428-013-0390-6 10.3758/s13428-013-0390-6 24142834

[CR28] Kruskal J (1964). Multidimensional scaling by optimizing goodness of fit to a nonmetric hypothesis. Psychometrika.

[CR29] Kruskal J (1964). Nonmetric multidimensional scaling: A numerical method. Psychometrika.

[CR30] Lingnau A, Schwarzbach J, Vorberg D (2008). Adaptive strategies for reading with a forced retinal location. Journal of Vision.

[CR31] Lingnau A, Schwarzbach J, Vorberg D (2010). (Un-) Coupling gaze and attention outside central vision. Journal of Vision.

[CR32] Manohar, S. G., & Husain, M. (2013). Attention as foraging for information and value. *Frontiers in Human Neuroscience*, *7*. 10.3389/fnhum.2013.00711 10.3389/fnhum.2013.00711 PMC3817627 24204335

[CR33] Mathôt S, Schreij D, Theeuwes J (2012). OpenSesame: An open-source, graphical experiment builder for the social sciences. Behavior Research Methods.

[CR34] McConkie GW, Rayner K (1975). The span of the effective stimulus during a fixation in reading. Perception & Psychophysics.

[CR35] McInnes, L., Healy, J., & Melville, J. (2018). UMAP: Uniform Manifold Approximation and Projection for Dimension Reduction. *ArXiv:1802.03426 [Cs, Stat]*. http://arxiv.org/abs/1802.03426. Accessed 20 Feb 2020

[CR36] Meng C, Zhao X (2017). Webcam-Based Eye Movement Analysis Using CNN. IEEE Access.

[CR37] Mills M, Dalmaijer ES, Van der Stigchel S, Dodd MD (2015). Effects of task and task-switching on temporal inhibition of return, facilitation of return, and saccadic momentum during scene viewing. Journal of Experimental Psychology: Human Perception and Performance.

[CR38] Mogg K, Millar N, Bradley BP (2000). Biases in eye movements to threatening facial expressions in generalized anxiety disorder and depressive disorder. Journal of Abnormal Psychology.

[CR39] Muhammed K, Dalmaijer ES, Manohar SG, Husain M (2020). Voluntary modulation of saccadic peak velocity associated with individual differences in motivation. Cortex.

[CR40] Mulckhuyse M, Dalmaijer ES (2016). Distracted by danger: Temporal and spatial dynamics of visual selection in the presence of threat. Cognitive, Affective, & Behavioral Neuroscience.

[CR41] Nord CL, Dalmaijer ES, Armstrong T, Baker K, Dalgleish T (2021). A causal role for gastric rhythm in human disgust avoidance. Current Biology.

[CR42] Papoutsaki, A., Sangkloy, P., Laskey, J., Daskalova, N., Huang, J., & Hays, J. (2015). WebGazer: Scalable Webcam Eye Tracking Using User Interactions. *Proceedings of the 33rd Annual ACM Conference Extended Abstracts on Human Factors in Computing Systems*, 219–222.

[CR43] Peirce J, Gray JR, Simpson S, MacAskill M, Höchenberger R, Sogo H, Kastman E, Lindeløv JK (2019). PsychoPy2: Experiments in behavior made easy. Behavior Research Methods.

[CR44] Rayner K (2014). The gaze-contingent moving window in reading: Development and review. Visual Cognition.

[CR45] Reisfeld D, Wolfson H, Yeshurun Y (1995). Context-free attentional operators: The generalized symmetry transform. International Journal of Computer Vision.

[CR46] Saragih JM, Lucey S, Cohn JF (2011). Deformable Model Fitting by Regularized Landmark Mean-Shift. International Journal of Computer Vision.

[CR47] Semmelmann K, Weigelt S (2018). Online webcam-based eye tracking in cognitive science: A first look. Behavior Research Methods.

[CR48] Teller DY (1979). The forced-choice preferential looking procedure: A psychophysical technique for use with human infants. Infant Behavior and Development.

[CR49] Theeuwes J, Kramer AE, Hahn S, Irwin DE (1998). Our eyes do not always go where we want them to go: Capture of the eyes by new objects. Psychological Science.

[CR50] Van der Stigchel S, de Vries JP (2015). There is no attentional global effect: Attentional shifts are independent of the saccade endpoint. Journal of Vision.

[CR51] Van der Stigchel S, Meeter M, Theeuwes J (2006). Eye movement trajectories and what they tell us. Neuroscience & Biobehavioral Reviews.

[CR52] Wagenmakers E-J (2007). A practical solution to the pervasive problems of *p* values. Psychonomic Bulletin & Review.

[CR53] Xu, P., Ehinger, K. A., Zhang, Y., Finkelstein, A., Kulkarni, S. R., & Xiao, J. (2015). TurkerGaze: Crowdsourcing Saliency with Webcam based Eye Tracking. *ArXiv:1504.06755 [Cs]*. http://arxiv.org/abs/1504.06755. Accessed 10 Feb 2021

[CR54] Yang, X., & Krajbich, I. (2020). *Webcam-based online eye-tracking for behavioral research* [Preprint]. PsyArXiv. 10.31234/osf.io/qhme6

