# Supplementary material for: MouseView.js: Reliable and valid attention tracking in web-based experiments using a cursor-directed aperture
Source: Behav Res Methods. 2021 Sep 29;54(4):1663–87. doi: 10.3758/s13428-021-01703-5 (PMC8480466; doi:10.3758/s13428-021-01703-5)

Supplementary Materials

MouseView.js: Reliable and valid attention tracking in web-based experiments using a cursor-directed aperture

Alexander L. Anwyl-Irvine [1], Thomas Armstrong [2]*, & Edwin S. Dalmaijer [1]*

1. MRC Cognition and Brain Sciences Unit, University of Cambridge, Cambridge, UK
2. Department of Psychology, Whitman College, Walla Walla, WA, USA

** Senior authors contributed equally*

Contact details
Alexander L. Anwyl-Irvine, MRC Cognition and Brain Sciences Unit, 15 Chaucer Road, Cambridge, CB2 7EF, United Kingdom.
Alexander.Irvine@mrc-cbu.cam.ac.uk

Note on image quality
The images used in the figures included herein were heavy blurred and further obscured by heatmaps. As a consequence, they no longer bear any resemblance to the original images, although they do still provide some information on the general saliency. This abides by the license of the images, which prohibits publication. However, it also leaves only a vague imprint of the saliency of the original images, which we admit does not look particularly appealing, but we nevertheless hope it provides sufficient context for the superimposed heatmaps.

disgust1

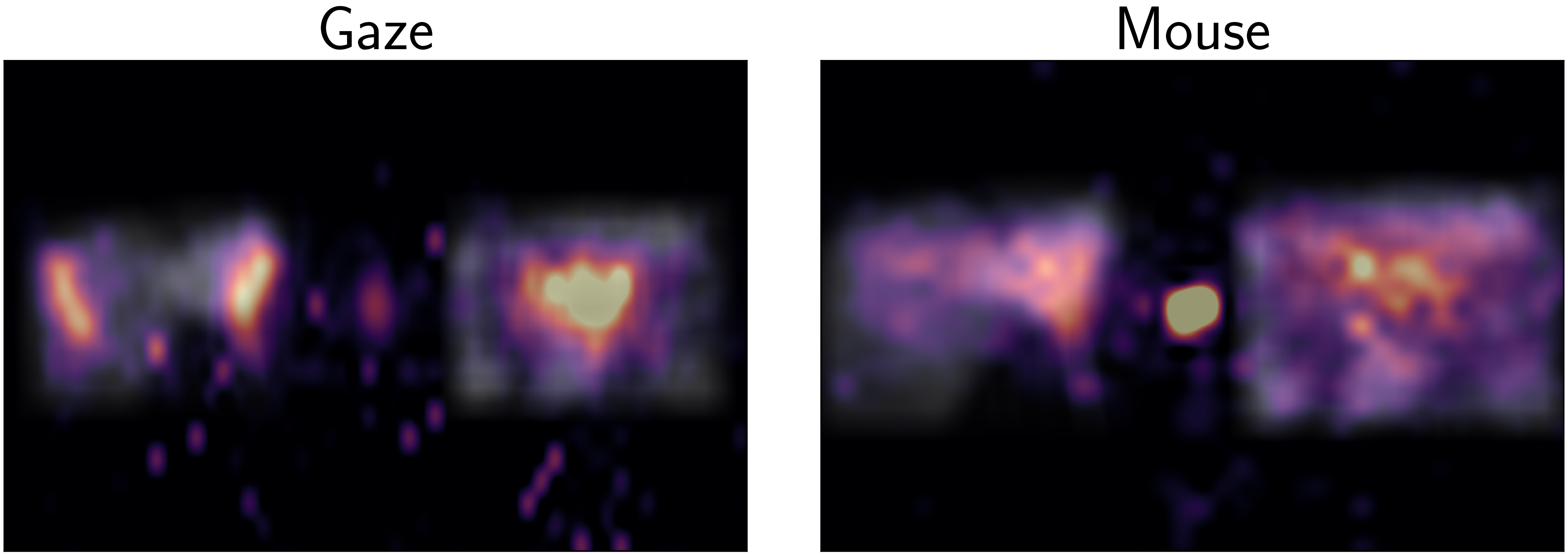

disgust2

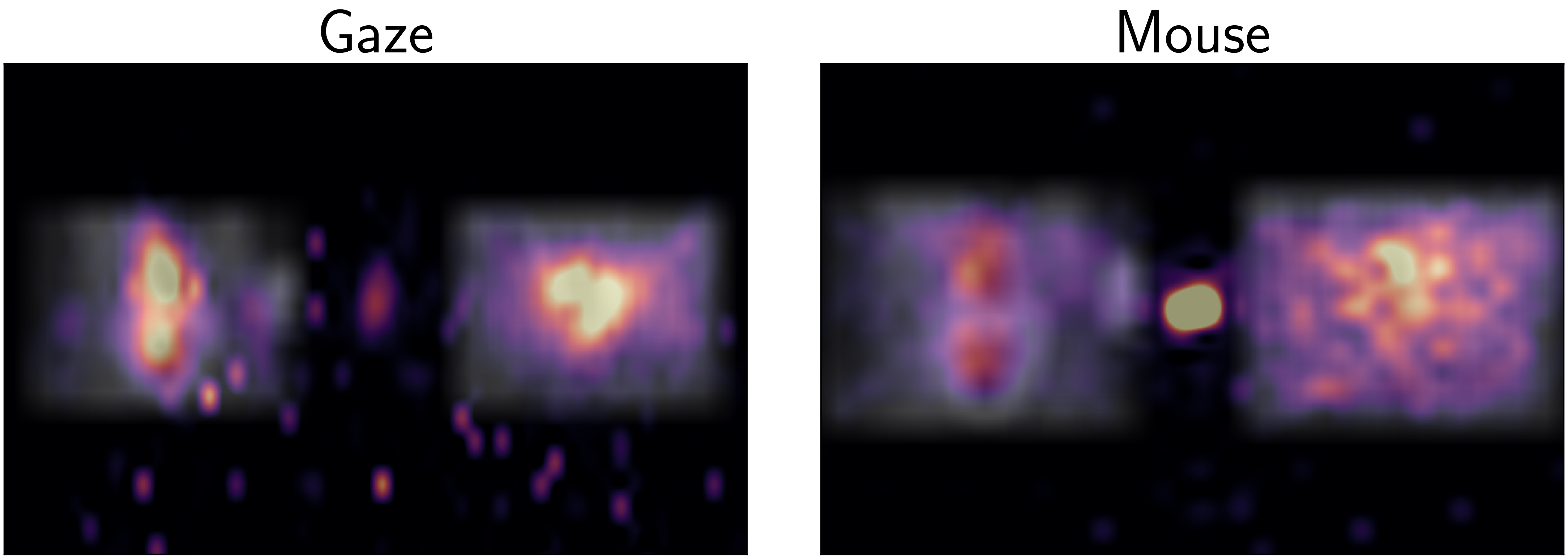

disgust3

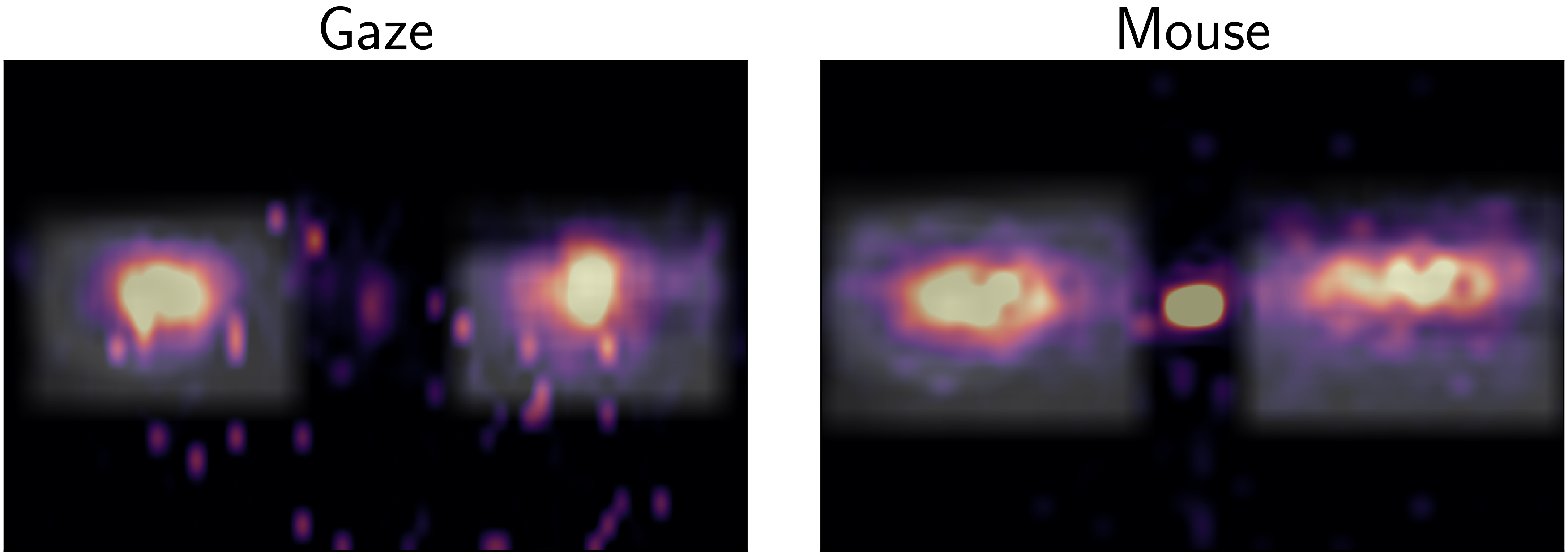

disgust4

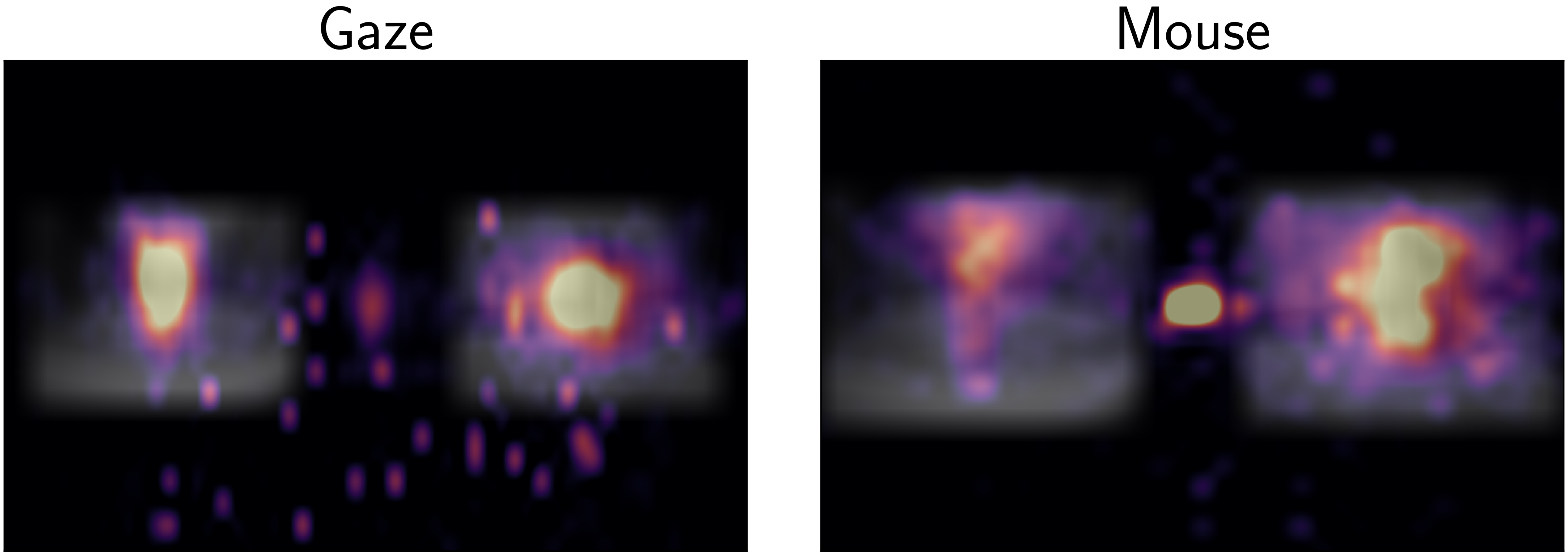

disgust5

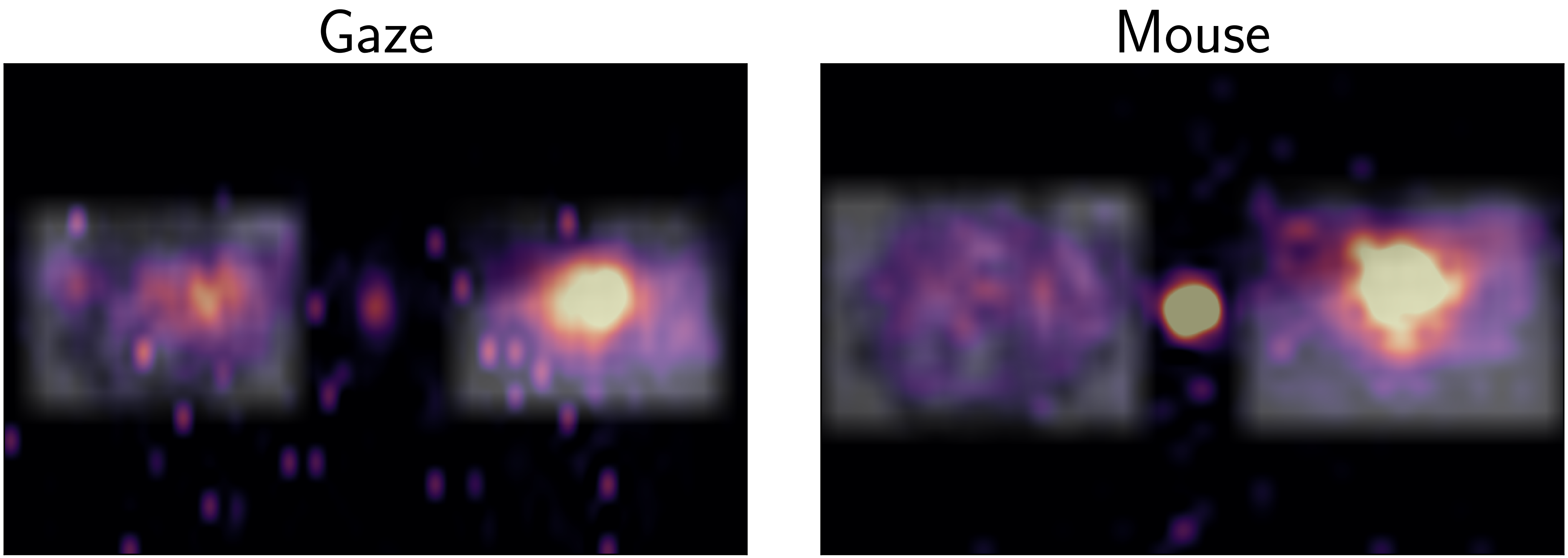

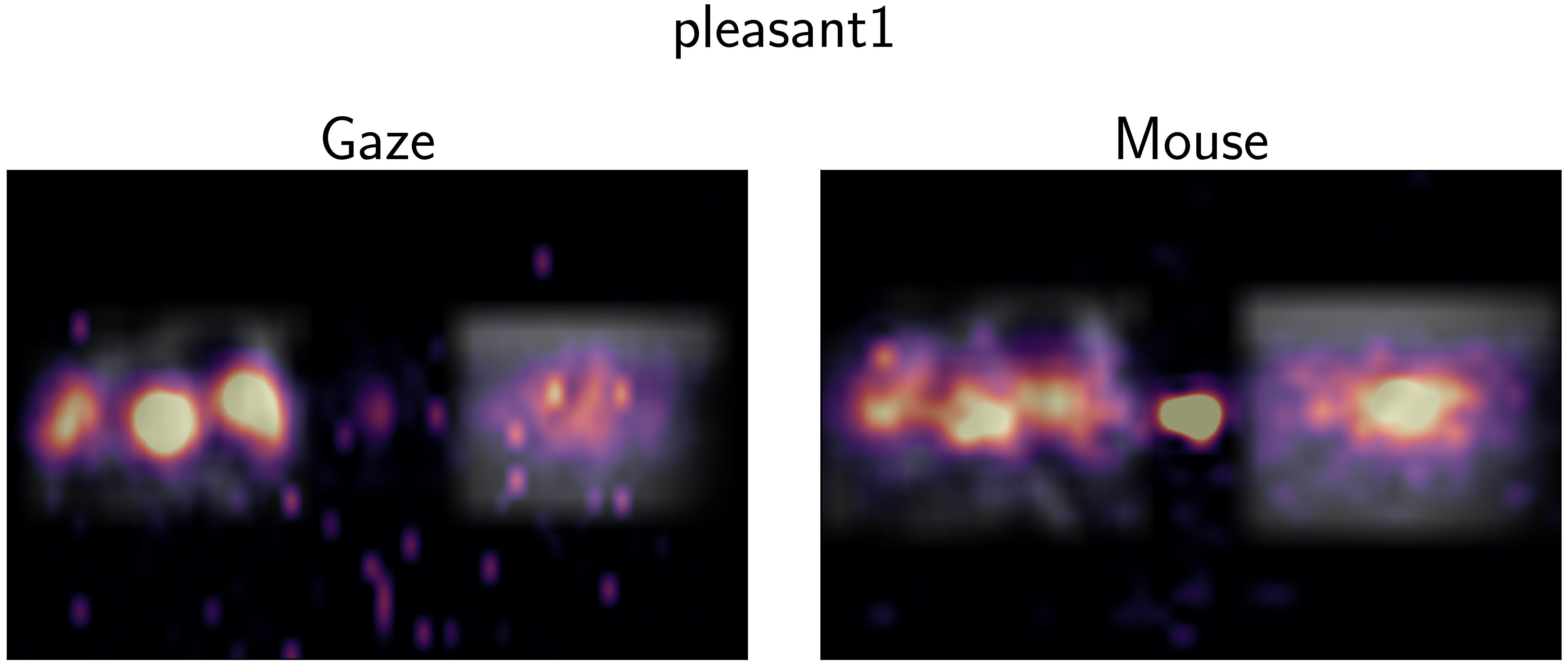
pleasant1
Gaze
Mouse

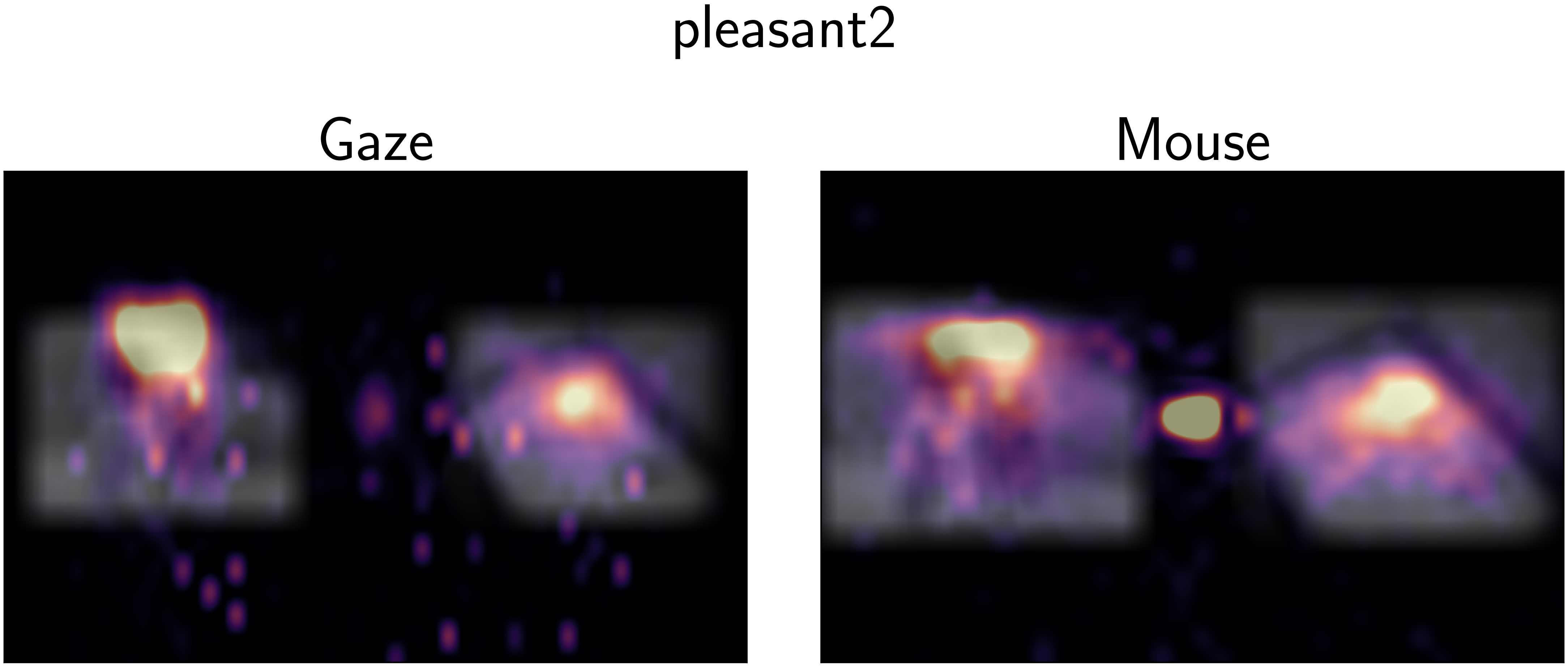
pleasant2
Gaze
Mouse

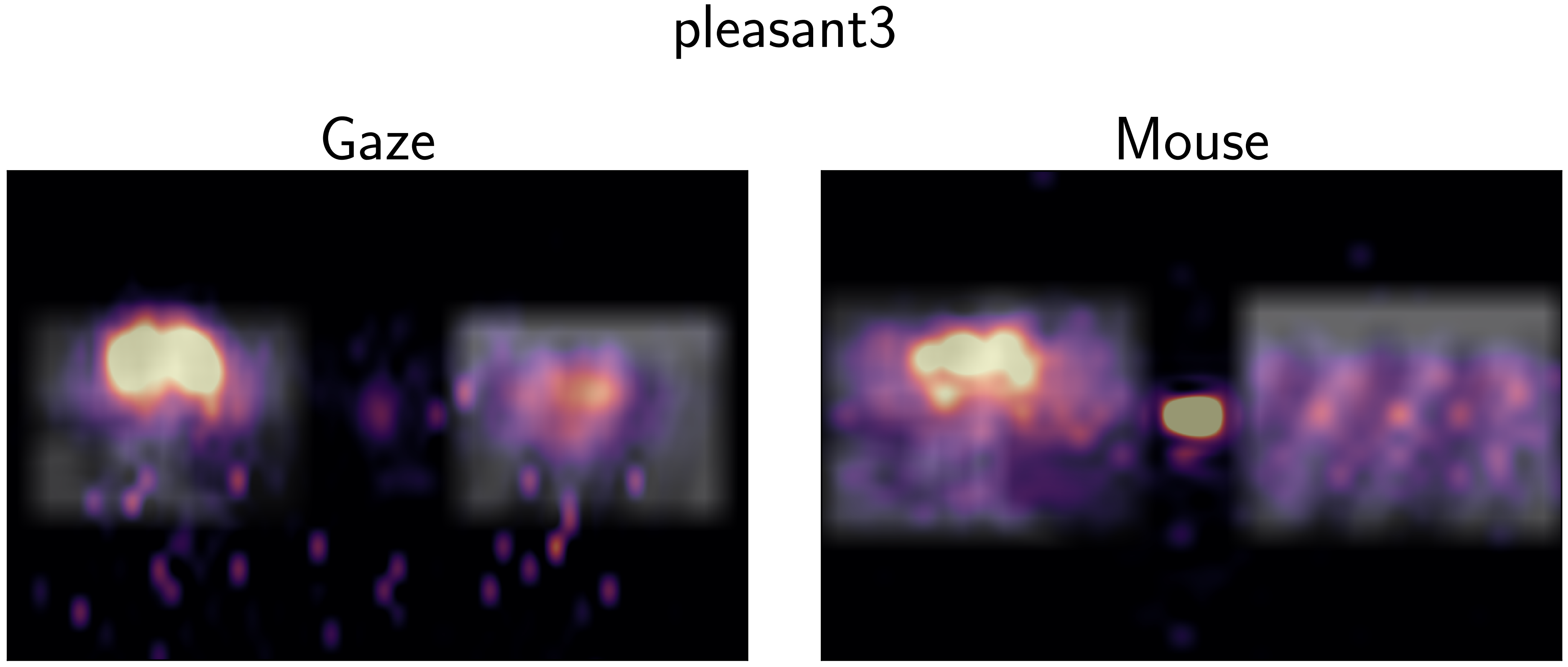
pleasant3
Gaze
Mouse

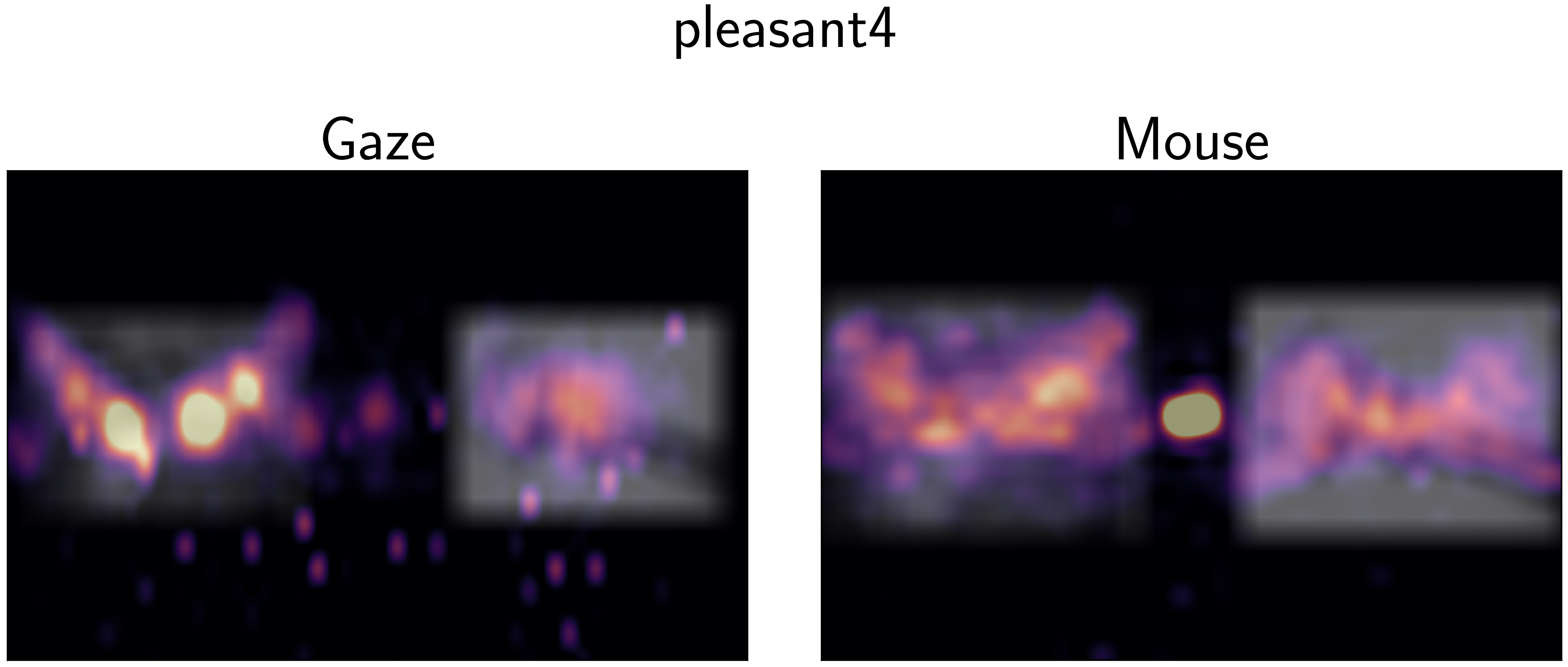
pleasant4
Gaze
Mouse

pleasant5

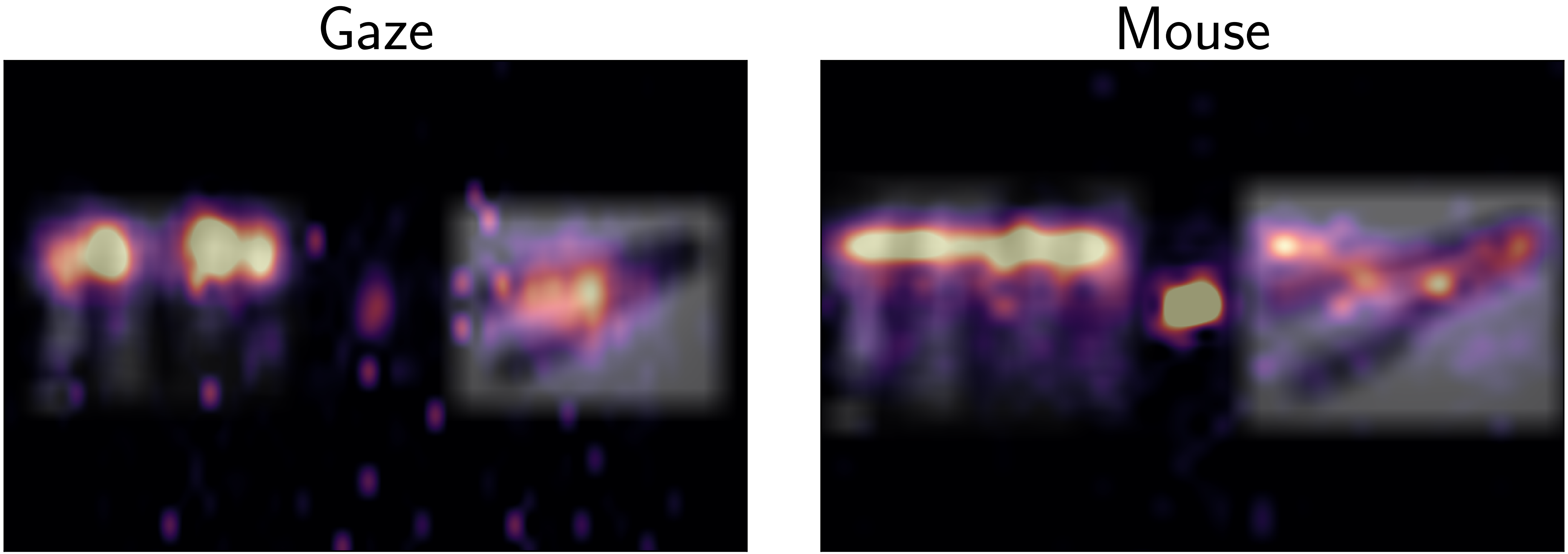

Supplement: Supplementary file 1 — (PDF 7215 kb) [file 13428_2021_1703_MOESM1_ESM.pdf]
